# Testing the stability of behavioural coping style across stress contexts in the Trinidadian guppy

**DOI:** 10.1111/1365-2435.12981

**Published:** 2017-09-24

**Authors:** Thomas M. Houslay, Maddalena Vierbuchen, Andrew J. Grimmer, Andrew J. Young, Alastair J. Wilson

**Affiliations:** ^1^ Centre for Ecology and Conservation University of Exeter Penryn Cornwall UK; ^2^ School of Biological & Marine Sciences Plymouth University Devon UK

**Keywords:** animal personality, behavioural syndromes, coping styles, individual‐by‐environment interactions, individual differences, individual plasticity, multiresponse model, *Poecilia reticulata*

## Abstract

1. Within populations, individuals can vary in stress response, a multivariate phenomenon comprising neuroendocrine, physiological and behavioural traits.
2. Verbal models of individual stress “coping style” have proposed that the behavioural component of this variation can be described as a single axis, with each individual's coping style being consistent across time and stress contexts.
3. Focusing on this behavioural component of stress response and combining repeated measures of multiple traits with a novel multivariate modelling framework, we test for the existence of coping style variation and assess its stability across contexts in the Trinidadian guppy (*Poecilia reticulata*).
4. Specifically, we test the following hypotheses: (1) there exists repeatable among‐individual behavioural (co)variation (“personality”) within a mild stress context consistent with a risk‐averse–risk‐prone continuum of behavioural coping style, (2) there is population‐level plasticity in behaviour as a function of stressor severity, (3) there is among‐individual variation in plasticity (i.e. IxE), and (4) the presence of IxE reduces cross‐context stability of behavioural coping style.
5. We found significant repeatable among‐individual behavioural (co)variation in the mild stress context (open field trial), represented as an **I** matrix. However, **I** was not readily described by a simple risk‐averse–risk‐prone continuum as posited by the original coping style model. We also found strong evidence for population‐level changes in mean behaviour with increasing stressor severity (simulated avian and piscine predation risks).
6. Single‐trait analyses did show the presence of individual‐by‐environment interactions (IxE), as among‐individual cross‐context correlations were significantly less than +1. However, multitrait analysis revealed the consequences of this plasticity variation were minimal. Specifically, we found little evidence for changes in the structure of **I** between mild and moderate stress contexts overall, and only minor changes between the two moderate contexts (avian vs. piscine predator).
7. We show that a multivariate approach to assessing changes in among‐individual (co)variance across contexts can prevent the over‐interpretation of statistically significant, but small, individual‐by‐environment effects. While behavioural flexibility enables populations (and individuals) to respond rapidly to changes in the environment, multivariate personality structure can be conserved strongly across such contexts.

A plain language summary is available for this article.

## 1 INTRODUCTION

Coping with challenging environments and situations is a necessary part of life. In vertebrates, overcoming these challenges and maintaining organismal function involves a complex suite of neuroendocrine, physiological and behavioural traits that together comprise the stress response (McEwen & Wingfield, 2010; Øverli et al., 2007; Romero, Dickens, & Cyr, 2009; Wingfield, 2003). Within populations, there can exist consistent differences among individuals in their stress response, spanning a continuum of “stress coping styles” (Koolhaas, 2008; Koolhaas, de Boer, Buwalda, & Van Reenen, 2007; Koolhaas et al., 1999). We propose that recent advances in the fields of animal personality and individual plasticity variation can provide a useful framework for testing hypotheses about the structure of the behavioural component of coping style variation and the extent to which it is consistent across multiple stress contexts. Here, we illustrate this framework empirically in a study of behavioural variation within and across stress contexts in the Trinidadian guppy, *Poecilia reticulata*.

The common verbal model of coping styles postulates that among‐individual variation will span a continuum from “reactive” to “proactive,” along which behavioural and physiological traits are predicted to both vary and covary in an integrated fashion (Koolhaas et al., 1999). To the extent that the nature of the stress response does differ among individuals, the behavioural components of coping style can be viewed as part of the broader phenomenon of animal “personality” (Réale, Dingemanse, Kazem, & Wright, 2010). While some have argued that personality predicts individual response to risks (Quinn, Cole, Bates, Payne, & Cresswell, 2012), others have treated coping style as a personality trait in its own right (Carere, Caramaschi, & Fawcett, 2010; Réale, Reader, Sol, McDougall, & Dingemanse, 2007). Although the distinction may be largely semantic, for current purposes, we adopt the latter position, noting that the coping style model posits “reactive–proactive” or “risk‐averse–risk‐prone” behavioural variation among individuals analogous to a “shy–bold” personality axis (see Boulton et al., 2015). This follows the common definition of boldness as an underlying axis of repeatable behavioural responses to perceived risk (Wilson, Clark, Coleman, & Dearstyne, 1994). Empirically, individuals are most commonly placed along a shy–bold axis using data from repeated behavioural observations (ideally of multiple traits, e.g. Boulton, Pearce, Wilson, Sinderman, & Earley, 2012; Carter & Feeney, 2012; White, Kells, & Wilson, 2016).

In this study, we focus on characterising variation in stress‐related behaviour in a captive population of the Trinidadian guppy (*P. reticulata*). We first characterise “behavioural coping style” via a multivariate description of movement patterns using modified open field trials (OFTs), a technique used widely with small fishes, including guppies (e.g. Burns, 2008; Smith & Blumstein, 2010; Warren & Callaghan, 1975; White et al., 2016). Since the OFT involves handling, transfer to a novel environment and isolation, we consider it a mild stressor for this shoaling species (Archard, Earley, Hanninen, & Braithwaite, 2012; Boulton et al., 2015). This enables us to test whether a single shy‐bold type axis of among‐individual behavioural variation provides an adequate model of repeatable behaviour across time within a single stress context.

**Figure 1:**
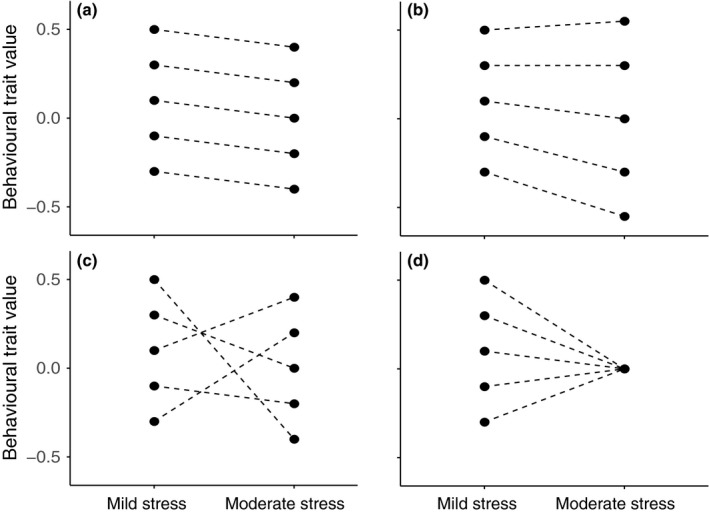
Examples of how variation in plasticity might affect the stability of “coping styles” across stress contexts. Each panel shows “coping style” behavioural variation (i.e. differences among individuals in their average behaviour) in the “mild stress” context (left hand side of *x*‐axis), with identical population‐level behavioural plasticity (the change in the mean behaviour across contexts). The four panels illustrate the outcome with no IxE (panel a) or three different forms of IxE (panels b–d): (a) coping styles are consistent across contexts (no IxE; V_mild_ == V_moderate_, cross‐context correlation *r* = 1); (b) increased stressor severity increases among‐individual behavioural variation (IxE), but rank order remains consistent (V_mild_  < V_moderate_, *r* = 1); (c) among‐individual variation exists within each context, but strong rank order changes (IxE) mean individual position cannot be predicted across contexts (V_mild_ == V_moderate_, *r* < 1); (d) all individuals converge on a common behaviour (IxE), such that there is actually no among‐individual variation in the moderate stress context (V_mild_ > 0, V_moderate_ = 0, *r* = 0)

However, the concept of a behavioural coping style is more compelling (and potentially more useful) if an individual's behavioural responses to stress are also consistent (and thus predictable) across stress contexts. Although personality studies emphasise the importance of among‐individual differences in mean behaviour, there is a growing appreciation that this can exist alongside among‐individual differences in behavioural plasticity (i.e. individual variation in the mean change in behaviour across contexts; Japyassú & Malange, 2014). Critically, such individual variation in plasticity, also known as individual‐by‐environment interaction or IxE (Alonzo, 2015; Dingemanse & Wolf, 2013; Mathot, Wright, Kempenaers, & Dingemanse, 2012; Stamps, 2016), is expected to erode cross‐context consistency in behaviour (and hence in behavioural coping styles; Figure 1). We therefore test for IxE and characterise the repeatable components of multivariate behaviour under two different moderate stressor contexts (visual cues of a fish predator, and both visual and disturbance cues of an avian predator strike), for comparison to the mild (OFT) context.

Guppies are a well‐known model for behavioural studies, particularly in relation to environmental stressors associated with predation risk. The species is known to exhibit strong behavioural responses to perceived risk of attack from aquatic and aerial predators (Templeton & Shriner, 2004), and previous research has shown guppy behaviours are repeatable under simple testing paradigms (Burns, 2008). Personality variation has been linked to predation risk (Harris, Ramnarine, Smith, & Pettersson, 2010), and Endler (1995) found among‐population variation in behaviour (as well as life history) associated with differences in predation regime. There is now evidence to suggest that such inter‐individual behavioural differences have both developmental (Fischer, Ghalambor, & Hoke, 2016) and genetic origins (Bleakley, Martell, & Brodie, 2006). Indeed, prior work on the population used here has shown repeatable variation in OFT traits consistent with underlying personality variation (White et al., 2016) that is partly driven by heritable (genetic) effects (S.J. White, unpublished data).

Our primary goals are to test the hypotheses that (1) there exists repeatable among‐individual behavioural (co)variation (“personality”) within a given context (OFT), consistent with a risk‐averse–risk‐prone (or shy–bold) continuum of behavioural coping style, (2) there is population‐level plasticity in behaviour as a function of stressor severity (i.e. differences in population‐level mean behaviour between the mild and moderate stress contexts), (3) there exists among‐individual variation in the nature of the plastic response to this change in stressor severity (i.e. IxE), and (4) the existence of such IxE causes a lack of cross‐context stability in behavioural coping style (manifest in significant changes across contexts in the overall among‐individual behavioural variation and/or between‐trait correlations). We use mixed effects models with repeated measures data to partition the among‐individual effects from within‐individual variation in each stress context. Since the use of single traits to infer personality axes can be problematic (Carter & Feeney, 2012; Carter, Feeney, Marshall, Cowlishaw, & Heinsohn, 2013; Wilson, de Boer, Arnott, & Grimmer, 2011), we employ a multitrait (and thus a multivariate analytical) approach. While the use of “reaction norm” models to study behavioural IxE has been strongly advocated in recent years (Dingemanse & Dochtermann, 2013; Dingemanse, Kazem, Réale, & Wright, 2010), this is (at least in our view) not ideal for multitrait analyses or for when individuals are assayed in more than two discrete environmental contexts, such that their relative positions on a continuous *x*‐axis are unknown (Houslay & Wilson, 2017). A secondary goal of our study is therefore to demonstrate a “character state” approach to multivariate IxE that has broad applicability beyond the current investigation of behavioural coping style.

## 2 MATERIALS AND METHODS

### 2.1 Husbandry

We used 128 sexually mature guppies (evenly split across sexes), sampled haphazardly from our captive population housed at the University of Exeter's Penryn Campus. This population is descended from wild fish collected in 2008 from the lower Aripo River, Trinidad. This site is viewed as “high predation” under the high‐ vs. low‐predation paradigm used in the literature to characterise guppy populations (Seghers, 1974a, 1974b). We tagged fish for individual identification purposes using a coloured elastomer (Northwest Marine Technology, http://www.nmt.us/products/vie/vie.shtml) after sedation by immersion in buffered MS222 (0.1 g/L). Fish were housed in single‐sex groups of 8 during the study, and fed to satiation twice daily (8–10 a.m. and 4–6 p.m.) using commercial flake food and laboratory‐prepared *Artemia salina* nauplii. The behavioural trials were carried out in four experimental “blocks,” each lasting 4 weeks. For analysis, we retained data only from those 105 individuals (51 males and 54 females) that completed at least two trials in each of the predator stimulus assays.

### 2.2 Behavioural assays

Individual behaviour was assessed in a 20 × 30 × 20 cm tank, filled to a depth of 5 cm with room‐temperature water from the main supply (22°C), and containing a small shelter. The tank was lit from below using a light box, and screened with a cardboard casing to prevent external visual disturbance. We caught fish individually from their home tank using a dip net, examined them quickly for identification tags and placed them immediately into the centre of the tank. After allowing 30 s for acclimation, we filmed behaviour for 120 s using a Sunkwang C160 video camera with 6–60 mm manual focus lens suspended above the tank. At the end of this period, we saved this “pre‐predator” recording (equivalent to the standard open field trial, OFT, as described in White et al., 2016, but for a shorter time period and with a refuge in the arena as described below), then applied a predator stimulus (see below) immediately and filmed behaviour for a further 120 s. We then saved this second recording as “post‐predator.” At the end of the post‐predator recording, we returned the fish to its home tank.

We used two distinct types of predator stimulus: a simulated bird strike and visual reveal of a piscivorous cichlid in an adjoining tank. Each guppy was exposed to both predator stimuli four times each over a period of 4 weeks, resulting in a total of 16 recordings per individual: 8 × pre‐predator OFT, 4 × post‐bird strike and 4 × post‐cichlid reveal. To control for order effects, guppies were grouped by home tank to undergo either bird strike or cichlid reveal trials first. Predator types were then alternated and were never carried out on consecutive days (resulting in gaps of 2 days between trials within weeks, and 4–5 days between the second trial of any given week and the first trial of the subsequent week). The water was replaced between each group of guppies and individual order was randomised within groups. The bird strike consisted of swinging a counterweighted model heron head into the observation tank such that it struck the water, causing a physical disturbance to the tank, then removing the head immediately (as described in Boulton et al., 2015). By contrast, we revealed the cichlid predator by removing a visual divider between its tank and the observation tank; the cichlid was then visible for the duration of the “post‐predator” recording, but caused no physical disturbance to the observation tank.

We used the tracking software Viewer II (BiObserve) to extract behavioural data automatically from each recording. Note that we used a slightly different tank configuration for each of the two predator stimuli (Figure S1), but each comprised a shelter zone, an exposed zone, and one or more non‐exposed zone(s). Within each predator treatment, these zone layouts were also used for the corresponding pre‐predator behavioural trait definitions, such that changes from pre‐ to post‐predator could be determined accurately. We used the following behaviours, characterised from the 120 s videos, in our analyses: “Area” (the percentage of the total tank area that the fish visited during a recording, determined using a 1 cm × 1 cm grid superimposed over the entire tank by the tracking software), “Exposed” (duration spent in the exposed zone, in seconds), “Freezings” (number of times the fish's speed dropped below the minimum velocity threshold of 4 cm/s for at least 2.5 s), “Shelter” (duration spent in the shelter zone, seconds) and “Tracklength” (total distance travelled, cm). Behaviours were selected on the basis of their expected contribution to aspects of boldness and/or exploration, and that measurements were not autocorrelated. Our potential maximum number of behavioural measurements (given 5 behaviours measured in 128 fish in 16 total recordings) was 10240; due to mortalities during the period of data collection (note also that we removed the entire records of those individuals that failed to complete at least 2 trials of each assay type from the dataset), our final total was 8150.

#### 2.2.1 Control group

Since all fish experienced the mild stress stimulus (pre‐predator OFT) before the moderate (post‐predator) one, we used a separate control group to test for temporal changes in behaviour over the recording periods in the absence of predator stimuli. A total of 32 untagged adult male guppies from the same stock population were recorded for a single replicate in the same manner as above, but no predator stimulus was applied: in the bird strike setup, we simply took two consecutive 120 s recordings; for cichlid reveal, we removed the visual barrier to reveal an adjacent empty tank for the second recording. We used only males here as most of our mature females had entered a breeding experiment for a separate study at this time.

### 2.3 Statistical analyses

We analysed all data using linear mixed effect models in r version 3.3.2 (R Core Team, 2016). Visual inspection of residuals from all models suggested all behaviours conformed to the assumption of residual normality. Behavioural measurements were scaled to standard deviation units (calculated from all observations—i.e. including pre‐bird strike, pre‐cichlid reveal, post‐bird strike and post‐cichlid reveal) prior to analysis, enabling more meaningful comparison of effect sizes across traits and assisting multivariate model fitting (described below). In all models, continuous observed predictors fitted as fixed effects (e.g. time of day) were standardised by mean‐centring and scaling, putting them on a common scale and aiding the interpretation of main effects (Gelman & Hill, 2007; Schielzeth, 2010). Other continuous predictors were mean‐centred only (e.g. order, replicate). We compared nested models using likelihood ratio tests (LRTs), in which we estimated χ^2^_*n*DF_ as twice the difference in model log likelihoods, with the number of degrees of freedom (*n*) equal to the number of additional parameters in the more complex model. When testing a single random effect, we assumed the test statistic to be asymptotically distributed as an equal mix of χ^2^_0_ and χ^2^_1_ (denoted as χ^2^_0,1_; Visscher, 2006). Except where explicitly noted below in relation to testing for population‐level mean response to stressor severity, fixed effects were used in our mixed models as statistical controls only; these are justified and described below in relation to models fitted, but estimates and their associated *p*‐values are reported only in supplemental materials if not relevant to the biological hypotheses being tested (Tables S1 and S2).

#### 2.3.1 Among‐individual behavioural (co)variation under mild stress

Using the observations from the pre‐predator portion of all trials, we fitted a series of nested models in ASreml‐R 3.0 (Butler, 2009) to partition multivariate behavioural variation into a between‐individual covariance matrix (subsequently denoted **I**_**pre**_) and a corresponding within‐individual (i.e. residual) component. Each model included trait‐specific fixed effects to control for effects not directly relevant to hypotheses being tested. These included *sex*, the *order* that individuals were assayed within a single tank of water (to allow for possible effects of water‐borne cues from previous fish), the *time* that the trial started (as seconds calculated from 9 a.m. each day) and *replicate* (i.e. cumulative total of trials experienced by an individual). Each model also included trait‐specific fixed effects of tank and experimental block. Since the tank configuration differed slightly between the two predator contexts, we also included a trait‐specific fixed effect of pre‐predator context (i.e. pre‐bird strike vs. pre‐cichlid reveal). Note that pooling data from the two trial types means that estimated **I**_**pre**_ will represent an average of variance–covariance structures from the two tank configurations if they differ. However, preliminary (univariate) models found no significant differences in among‐individual variance (*V*_I_) between pre‐bird strike and pre‐cichlid reveal trials for any trait, and among‐individual correlations (*r*_I_) across these configurations were not significantly different from +1 (all *p *>* *.35).

Our nested models featured different covariance specifications to test the expectation that there would be among‐individual variance and covariance structure consistent with the presence of an axis of boldness variation. **Model 1A** has no random effects, such that all phenotypic variance (conditional on the fixed effects) is allocated to the residual component **R** (which can be considered “within‐individual” here). We specified **R** as a “diagonal” matrix, where variances for each behavioural trait are estimated but all among‐trait covariance terms are set to zero. **Model 1B** includes individual ID as a random effect, with among‐individual component **I** also specified as a diagonal matrix. **Model 1C** allows among‐trait covariance in **R** (i.e. estimating the off‐diagonals in the residual covariance matrix). **Model 1D** extends **1C** by also allowing among‐trait covariance in **I**. We then used likelihood ratio tests to provide global tests (i.e. across all traits) for (1) among‐individual behavioural variation (1A vs. 1B), (2) among‐trait covariation (1B vs. 1C) and (3) significant contribution of individual differences to this among‐trait covariation (1C vs. 1D). Our final estimates of **I**_**pre**_ and **R**_**pre**_ are based on **Model 1D** (i.e. the fully unconstrained model). Note that since behaviours were scaled to standard deviation units (from all measurements across stages and contexts) prior to analysis, the among‐individual variance (*V*_I_) terms on the diagonal of **I**_**pre**_ can be viewed as analogous to repeatabilities (since repeatability = *V*_I_/*V*_P_, and the observed phenotypic variance *V*_P_ is 1). We also estimated the adjusted repeatability of each behaviour within‐context (where *V*_P_ in this case is the sum of among‐individual and residual variance from a context‐specific model, having conditioned on fixed effects). We repeated these procedures using data from the post‐bird strike (**Models 2A–2D**) and post‐cichlid reveal (**Models 3A–3D**), such that models **2D** and **3D** yield estimates of **I**_**post‐bird**_ and **I**_**post‐cichlid**_. The inclusion of sex as a fixed effect in all models means that the among‐individual (co)variance estimates (and comparisons thereof) are thus estimates of (co)variance around sex‐specific means. We therefore assume homogeneity of **I** matrices across sexes, or—equivalently—we estimate **I** matrices that are interpretable as being averaged across any sex differences.

To aid the interpretation of covariance terms contained in **I**_**pre**_, we calculated the corresponding among‐individual correlations r_Ipre_ (where for any pair of traits (x,y), *r*_Ipre(x,y)_ = COV_Ipre(x,y)_/(√(*V*_Ipre(x)_) × √(*V*_Ipre(y)_)). We also subjected **I**_**pre**_ to eigen decomposition to determine the proportion of among‐individual variation captured by each principal component. We used this eigen decomposition to assess whether a single major axis of variation could indeed explain most of the among‐individual variation (consistent with the simple proactive‐reactive coping style model). We estimated uncertainty on the trait loadings associated with each principal component (eigen vector) using the parametric bootstrap approach as described by Boulton, Grimmer, Rosenthal, Walling, and Wilson (2014).

#### 2.3.2 Population‐level response to increased stressor severity

To test for population‐level (i.e. mean individual) plasticity in each behavioural trait as a function of stressor severity, we fitted univariate mixed models in the r package lme4 (Bates, Mächler, Bolker, & Walker, 2015). We fitted separate models for each behaviour with each predator type, but using data from both the pre‐ and post‐predator stages of the trial. A fixed effect of *stage* (i.e. pre‐ vs. post‐predator stimulus, coded as −0.5 and 0.5, respectively) was modelled to test for a change in behaviour with increased stressor severity. Additional fixed effects included the *time of day* at which the OFT started (in seconds, mean‐centred and scaled), as well as *sex*,* order* and *replicate* (as described above). Random effects were tank, experimental block and individual ID. For each combination of behaviour and predator type, we used a likelihood ratio test to compare this model (fitted using ML) to one without the *stage* predictor.

We also used data from the control group to check whether apparent effects of *stage* might be driven by a temporal confound (rather than the predator stimulus *per se*). We used similar univariate mixed models as for the data for testing stressor severity, but with fixed effects only of *stage* and *order* (as assay‐specific controls were run from males selected from a single tank on a single day, such that *replicate*,* tank* and *block* were not required; we also omitted *time of day* as it was highly correlated with *order*). Individual ID was fitted as a random effect. For this smaller dataset, some transformations were required in order that residuals met the assumptions of normality for all behaviours (namely, square‐root transformation for duration exposed and number of freezings and log + 1 transformation for duration in the shelter zone, in the “cichlid presence” setup only). We used a likelihood ratio test to compare the full model (fitted using ML) to one without the *stage* predictor to test whether mean fish behaviours changed across stages in the absence of the predator.

#### 2.3.3 IxE: Among‐individual variance in behavioural plasticity

Finally, we tested for among‐individual variation in behavioural plasticity (IxE) to increased stressor severity: significant IxE would indicate that individuals differ in the magnitude of their behavioural change across stress contexts. While variation in behavioural plasticity is most commonly modelled using reaction norms (Dingemanse & Dochtermann, 2013; Dingemanse et al., 2010), this framework is only applicable to more than two environments (here stress contexts) if they can be placed on a continuous axis (i.e. “function‐valued traits”; Stinchcombe & Kirkpatrick, 2012). In our study, we make no assumption about the relative severity of the two higher stress (post‐predator) contexts, rendering the reaction norm approach problematic (Brommer, 2013). Furthermore, while linear reaction norms allow an intuitive separation of the context‐dependent and ‐independent components of a trait (i.e. plasticity as slope, and mean phenotype as intercept), this interpretation does not scale readily to the multitrait case, where interpreting covariances between intercept and slope terms for different behavioural traits quickly becomes unintuitive (e.g. the covariance between the intercept for area covered and the slope for shelter use). We instead use a character state approach, which can (given enough data) be extended to any number of discrete environments, thus enabling estimation—and therefore direct comparison—of among‐individual variance in each context, in addition to all cross‐context covariances.

For a behavioural trait expressed in a given stress context, let fish *j* have an expected individual deviation (from the population mean) of *i*_*j*_. In the absence of IxE, this deviation—expressed relative to the context‐specific mean—is independent of the “environment” such that *i*_*j(pre‐predator)*_ = *i*_*j(post‐bird)*_ = *i*_*j(post‐cichlid)*_. It therefore follows that the variance in *i* (V_I_, the among‐individual variance in a given trait) is homogeneous across contexts. It also follows that the cross‐context correlation of individual deviation must equal +1. Put simply, a lack of IxE means that among‐individual variation remains the same across contexts, and that an individual's performance (relative to the phenotypic mean) in one context perfectly predicts its (relative) performance in another. Thus, for each behaviour separately, starting with “Area,” we defined three context‐specific response variables: pre‐predator (pooled across assay types), post‐bird strike and post‐cichlid reveal. We then used a series of bivariate models to estimate and test the three cross‐context correlations of individual deviations: *r*_*i(*pre, post‐bird)_, *r*_*i(*pre, post‐cichlid)_ and *r*_*i(*post‐bird, post‐cichlid)._ For each cross‐context combination, we fit models with the following constraints: the cross‐context correlation constrained to zero, correlation constrained to one, and unconstrained correlation (note that all correlation estimates were positive, so we did not create a model constrained to negative one). We used LRTs to test the unconstrained model against the zero model (i.e. is the correlation significantly different from zero, such that there is some level of positive correlation in individual performance across contexts?) and the perfect correlation model (is the correlation significantly less than one, such that there does exist some statistically significant variation in individual performance across contexts or IxE?). Fixed effects included context‐specific means and effects of sex, replicate, order and time, in addition to overall effects of tank and experimental block. A separate mean was also included for each assay type in the pooled pre‐predator context. This process was repeated for the remaining behavioural variables (“Exposed,” “Freezings,” “Shelter” and “Tracklength”).

Extending the above to the multi‐trait case, an absence of IxE means that **I**_**pre**_ = **I**_**post‐bird**_ = **I**_**post‐cichlid**_. Similarity (or lack thereof) between matrices can be assessed in many ways (e.g. Melo, Garcia, Hubbe, Assis, & Marroig, 2015; Roff, Prokkola, Krams, & Rantala, 2012), and here we used two complementary approaches (noting that all behavioural observations were scaled by their global standard deviation prior to analysis, putting each type of trait on a common scale but conserving any differences across contexts). First, we compared the traces (sum of diagonal elements) to determine simply where the total among‐individual behavioural variance differed between contexts. Second, we calculated “difference matrices” (**D**) between pairs of **I**, simply by subtracting one matrix from another (e.g. **D**_pre:post‐bird_ = **I**_post‐bird_ − **I**_pre_). Noting that if **I** matrices are identical then all elements of **D** will equal zero, we used parametric bootstrapping to estimate 95% confidence intervals around each element (and also on our trace comparisons). While this allows statistical inferences to be made, we caution that the confidence intervals estimated are necessarily approximate and based on assumed multivariate normality (see Boulton et al., 2014; Houle & Meyer, 2015 for discussion). We provide R code for this bootstrapping approach in Appendix S1.

## 3 RESULTS

### 3.1 Among‐individual behavioural (co)variation under mild stress

In the pooled “pre‐predator” mild stress context, comparison of models 1A–1D provided evidence of significant among‐individual variance in multivariate phenotype, as well as covariance structure among traits driven in part by individual‐level effects (Table 1). Table 2a shows the among‐individual variance–covariance matrix **I**_pre_ estimated under Model 1D, in which the *V*_I_ estimates for each trait (analogous to behavioural repeatabilities over the full range of behaviours expressed in all contexts) are on the diagonal of the matrix. Table 3 shows the adjusted repeatabilities (i.e. repeatability calculated after controlling for confounding effects; Nakagawa & Schielzeth, 2010) estimated within each context, which are low to moderate overall (ranging from 0.13 to 0.3). Overall, we find evidence for significant among‐individual behavioural (co)variation (i.e. “personality”) under mild stress.

**Table 1:** Multivariate model comparisons showing tests of among‐individual variation, among‐trait covariance and among‐individual trait covariance within each context (pre‐predator, post‐bird strike and post‐cichlid reveal). Models were fitted as described in main text and compared by likelihood ratio test

Context	Comparison	Testing for	χ^2^	*df*	*p*
Pre‐predator	1A vs. 1B	Variance among individuals	394.9	5	<.001
	1B vs. 1C	Among‐trait covariance	1435.5	10	<.001
	1C vs. 1D	Among‐individual trait covariance	175.5	10	<.001
Post‐bird strike	1A vs. 1B	Variance among individuals	88.5	5	<.001
	1B vs. 1C	Among‐trait covariance	1366.2	10	<.001
	1C vs. 1D	Among‐individual trait covariance	51.0	10	<.001
Post‐cichlid reveal	1A vs. 1B	Variance among individuals	113.9	5	<.001
	1B vs. 1C	Among‐trait covariance	508.9	10	<.001
	1C vs. 1D	Among‐individual trait covariance	49.0	10	<.001

**Table 2:** Among‐individual (**I**) variance‐covariance matrices estimated from (a) pooled pre‐predator data, (b) post‐bird strike data and (c) post‐cichlid reveal data. Among‐individual variances (*V*_I_, analogous to repeatabilities over the full range of behavioural measurements) are given on the diagonals, with among‐individual between‐trait covariances (COV_I_) below and the corresponding correlations (*r*_I_) above. 95% confidence intervals in parentheses are based on 5,000 bootstrapped **I** matrices

	Area	Exposed	Freezings	Shelter	Tracklength
(a) Pre‐predator					
Area	0.18 (0.10, 0.27)	0.30 (−0.05, 0.62)	−0.14 (−0.47, 0.16)	−0.52 (−0.75, −0.26)	0.57 (0.33, 0.78)
Exposed	0.05 (−0.01, 0.11)	0.15 (0.08, 0.23)	0.83 (0.66, 0.99)	−0.80 (−0.96, −0.64)	−0.03 (−0.36, 0.31)
Freezings	−0.03 (−0.09, 0.03)	0.16 (0.08, 0.23)	0.24 (0.14, 0.33)	−0.59 (−0.81, −0.38)	−0.37 (−0.63, −0.11)
Shelter	−0.09 (−0.14, −0.03)	−0.12 (−0.18, −0.06)	−0.11 (−0.17, −0.05)	0.15 (0.09, 0.22)	−0.47 (−0.69, −0.22)
Tracklength	0.11 (0.04, 0.18)	−0.01 (−0.05, 0.06)	−0.08 (−0.14, −0.02)	−0.08 (−0.14, −0.03)	0.20 (0.12, 0.28)
(b) Post‐bird strike					
Area	0.09 (0.01, 0.16)	0.41 (−0.13, 0.82)	0.24 (−0.38, 0.82)	−0.59 (−1.00, −0.07)	0.57 (0.11, 0.90)
Exposed	0.06 (−0.02, 0.15)	0.29 (0.13, 0.44)	0.93 (0.79, 1.10)	−0.87 (−1.06, −0.67)	0.07 (−0.44, 0.44)
Freezings	0.03 (−0.04, 0.09)	0.21 (0.09, 0.32)	0.18 (0.07, 0.28)	−0.64 (−0.91, −0.28)	−0.21 (−0.72, 0.26)
Shelter	−0.07 (−0.14, 0.01)	−0.18 (−0.30, −0.07)	−0.11 (−0.20, −0.01)	0.16 (0.05, 0.27)	−0.50 (−0.85, −0.08)
Tracklength	0.06 (0.00, 0.12)	0.01 (−0.06, 0.08)	−0.03 (−0.09, 0.03)	−0.07 (−0.14, 0.00)	0.12 (0.05, 0.19)
(c) Post‐cichlid reveal					
Area	0.25 (0.13, 0.37)	0.52 (−0.01, 0.95)	0.12 (−0.27, 0.51)	−0.59 (−0.91, −0.26)	0.66 (0.40, 0.91)
Exposed	0.06 (0.00, 0.12)	0.06 (0.01, 0.12)	0.48 (−0.04, 0.97)	−0.81 (−1.37, −0.37)	0.56 (0.06, 1.14)
Freezings	0.03 (−0.07, 0.12)	0.06 (0.00, 0.13)	0.28 (0.14, 0.42)	−0.72 (−1.04, −0.43)	−0.22 (−0.63, 0.20)
Shelter	−0.12 (−0.20, −0.03)	−0.08 (−0.14, −0.02)	−0.15 (−0.26, −0.06)	0.16 (0.06, 0.27)	−0.45 (−0.81, −0.04)
Tracklength	0.14 (0.05, 0.22)	0.06 (0.00, 0.11)	−0.05 (−0.13, 0.04)	−0.08 (−0.15, 0.01)	0.17 (0.08, 0.28)

**Table 3:** Adjusted repeatabilities (estimate and *SE*) for each behaviour, calculated within each context

Behaviour	Pre‐predator	Post‐bird strike	Post‐cichlid reveal
Area	0.20 (0.04)	0.14 (0.05)	0.30 (0.06)
Exposed	0.17 (0.04)	0.26 (0.06)	0.13 (0.05)
Freezings	0.27 (0.04)	0.21 (0.06)	0.27 (0.06)
Shelter	0.27 (0.04)	0.17 (0.05)	0.18 (0.06)
Tracklength	0.27 (0.04)	0.22 (0.06)	0.23 (0.06)

### 3.2 No single major axis of among‐individual behavioural (co)variation

Examination of the between‐trait correlations in **I**_pre_ (*r*_I_; Table 2a, above‐diagonals) indicates a number of significant pairwise relationships, both positive and negative (correlations where 95% confidence intervals do not cross zero are considered nominally significant). However, the results of our eigen analysis were not consistent with a single major axis of variation in **I**_pre_; rather, the first 2 eigen vectors of **I**_**pre**_ both explained large amounts of among‐individual variation (EV1_pre_ = 49.7%, EV2_pre_ = 39.8%), accounting for almost 90% altogether. We did not therefore find a single major axis of among‐individual variation, as expected if observed behaviours are indicative of a single latent shy/bold (or reactive/proactive) axis as suggested by verbal models of behavioural coping styles.

**Figure 2:**
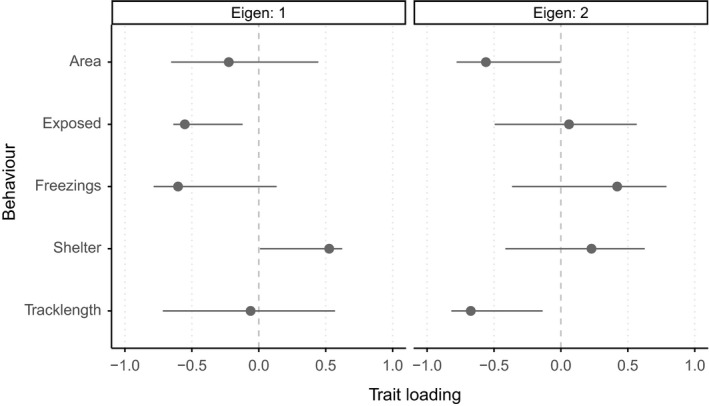
Trait loadings on the first two eigenvectors (eigen one, left; eigen two, right), from I_pre_ (the I matrix for pooled pre‐predator open field trial (OFT) behavioural variation). Lines represent 95% confidence intervals, calculated from 5000 bootstrapped replicates. Loadings are considered nominally significant if CIs do not cross zero (dashed vertical line). Arithmetic sign of loading denotes groups of behaviours that load in opposing directions (i.e. eigen one represents an axis where one extreme features individuals that spend more time in the exposed zone with a greater number of freezings and less time in the shelter; the other extreme those that spend greater time in the shelter, with fewer freezings and less time in the exposed zone)

For the first eigenvector EV1_pre_, exposed duration and number of freezings loaded strongly in the same direction, with shelter duration loading heavily in the other (Figure 2). Area covered and tracklength loaded in the same direction as exposed duration and number of freezings, but their estimates were close to zero (with large confidence intervals). EV2_pre_ loaded strongly on area covered and tracklength in one direction, and number of freezings in the other. The first axis suggests a behavioural decision regarding shelter use, while the second suggests alternative strategies for those finding themselves outside of the shelter.

### 3.3 Predator stimuli induce population‐level changes in behaviour

**Figure 3:**
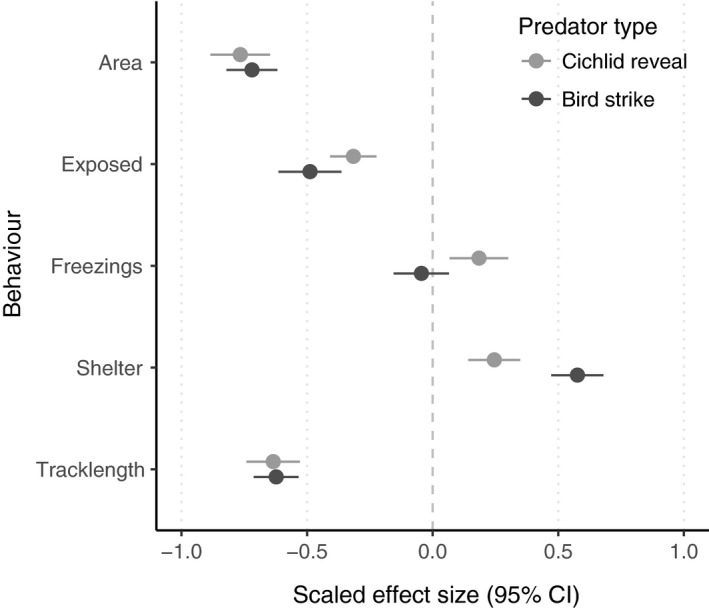
The estimated effect of predator stimulus (cichlid reveal, light grey; bird strike, dark grey) on average guppy behaviour. All behaviours (response variables) were mean‐centred and scaled to 1 *SD* for purposes of comparison. Effect sizes and confidence intervals (calculated as 1.96 times the *SE*) were taken from linear mixed model analyses (see text for details). Effects are considered nominally significant if CIs do not cross zero (dashed vertical line). Both predator stimuli induced significant population‐level plasticity in all behaviours (*p* = .002), except for the effect of bird strike on the number of freezings (*p* = .421)

Consistent with our prediction of population‐level plasticity in behaviour as a function of stressor severity, we found that both the bird strike and cichlid predator stimuli induced significant changes in the means of almost all behaviours (Figure 3). Both the bird strike and the cichlid reveal caused individuals to—on average—cover less area of the tank, travel less distance, spend less time in the exposed zone, and spend more time in the shelter (all *p *<* *.001). These results indicate a shift towards more putatively “shy” behavioural means in the higher stress (post‐predator) contexts than was observed in the lower stress (pre‐predator) context. The mean number of freezings presents a single exception to this general shift: freezings increased significantly after the cichlid reveal (*p *=* *.002), but saw a non‐significant decrease after the bird strike (*p *=* *.421).

In our control group, we found no significant effects of time stage for 9 of the 10 assay‐specific behavioural traits (Table S3). Total tracklength was reduced after removal of the visual barrier (to show adjacent empty tank) in the “cichlid reveal” assay setup (estimate = −71.25 ± 22.37, χ^2^_1_ = 8.8, *p *=* *.003). Given that this was the only behaviour affected significantly in this control context (where—unlike in the bird strike control—there was a physical change to the environment, the removal of the barrier), we therefore assume differences in the main experiment (as described above) are largely due to the predator stimuli.

### 3.4 Investigating IxE using trait‐specific tests

**Figure 4:**
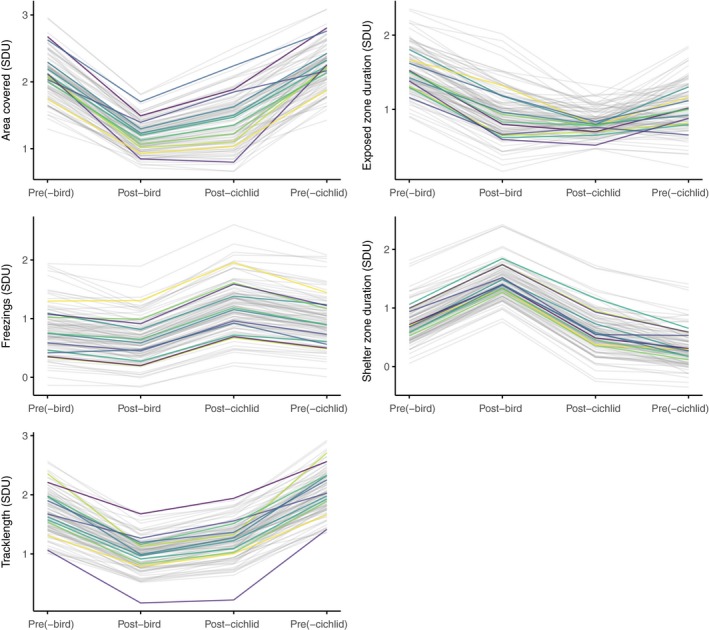
Each line shows an individual's intercept deviation (after conditioning on main effects) across pre‐predator, post‐bird strike and post‐cichlid reveal stages (“pre” is shown twice to enable easier comparison of changes across all stages). Deviations are estimated by multivariate models with pooled pre‐predator, post‐fish and post‐bird responses, with a separate model for each behaviour (see text for details). We use assay‐ and stage‐specific means to show both individual‐ and population‐level plasticity. We randomly selected eleven individuals (coloured lines) to illustrate reaction norms more clearly both within and across panels

Estimated cross‐context among‐individual correlations were significantly greater than zero for all behavioural traits and stress context pairs (Table 4). These cross‐context correlations and associated changes in among‐individual variance across contexts, are illustrated in Figure 4. For each behaviour in turn, we extracted individual BLUPs from trivariate models (with response variables being the behaviour in pooled pre, post‐bird strike and post‐cichlid reveal contexts), and added these to the assay‐ and stage‐specific population means (pre‐bird strike, pre‐cichlid presence, post‐bird strike and post‐cichlid presence) so as to illustrate changes in average behaviour as well as in among‐individual variation.

**Table 4:** Cross‐context among‐individual correlations for each behaviour, with tests of whether they are significantly different from 0 (i.e. positive correlation) and +1 (i.e. not perfect correlation). All correlations are significantly greater than 0. Correlations in bold are both significantly different from 0 and +1, indicating significant individual‐by‐environment interactions (IxE)

Behaviour	Contexts		Correlation	*SE*	Compare to 0		Compare to 1	
					χ^2^_1_	*p*	χ^2^_1_	*p*
Area	Pre	Post‐bird	.76	.15	16.1	<.001	2.4	.060
	Pre	Post‐cichlid	**.68**	.11	21.7	<.001	12.9	<.001
	Post‐bird	Post‐cichlid	.96	.16	26.4	<.001	−0.3	.500
Exposed	Pre	Post‐bird	**.77**	.11	19.4	<.001	4.3	.019
	Pre	Post‐cichlid	**.42**	.18	4.3	.019	7.0	.004
	Post‐bird	Post‐cichlid	**.57**	.20	5.0	.013	4.4	.018
Freezings	Pre	Post‐bird	**.83**	.08	36.8	<.001	4.7	.015
	Pre	Post‐cichlid	.91	.08	49.3	<.001	0.6	.220
	Post‐bird	Post‐cichlid	.94	.11	38.9	<.001	−0.1	.500
Shelter	Pre	Post‐bird	.92	.10	33.4	<.001	0.5	.237
	Pre	Post‐cichlid	**.78**	.09	32.0	<.001	9.2	.001
	Post‐bird	Post‐cichlid	.94	.14	25.3	<.001	−0.4	.500
Tracklength	Pre	Post‐bird	**.74**	.10	25.6	<.001	7.3	.003
	Pre	Post‐cichlid	**.69**	.11	22.0	<.001	11.5	<.001
	Post‐bird	Post‐cichlid	.85	.13	21.4	<.001	1.4	.115

For all traits, an individual's behaviour relative to the population mean in one stress context (e.g. area covered in the OFT prior to predator presentation) is therefore strongly predictive of its relative behaviour in other stress contexts (e.g. area covered following a predator presentation). However, 8 of the 15 correlations were also significantly less than +1 (Table 3). Of these, seven were between pre‐predator and post‐predator contexts, while the post‐bird—post‐cichlid correlation was only significantly less than +1 for a single behaviour (“Exposed”). Although this could reflect variation in power (sample sizes were larger for the pooled pre‐predator behaviours), there is also a pattern of higher correlations between the two higher stress (post‐predator) contexts (median across traits of 0.938) than between pre‐ and post‐predator contexts (median 0.765). We conclude from the existence of correlations significantly less than +1 that individual‐by‐environment interactions are occurring and—equivalently—that there is among‐individual variance in plasticity of behavioural response to stressor context. Notably, this IxE is largely occurring between the pre‐predator and post‐predator (i.e. mild and more severe) stress contexts and so is consistent with among‐individual variance in behavioural plasticity as a response to a change in the level of stress.

### 3.5 Investigating IxE by examining conservation of I matrix structure across contexts

Extending to the multitrait case, comparisons of Models 2A–D and 3A–D provided formal confirmation that fish in post‐bird and post‐cichlid contexts also exhibited significant among‐individual (co)variation in behavioural traits assayed (Table 1). Similarly to **I**_**pre**_, examination of between‐trait correlations in **I**_**post‐bird**_ and **I**_**post‐cichlid**_ (*r*_I_; Table 2b,c, above‐diagonals) indicates a number of significant pairwise relationships among the observed traits, both positive and negative. However, despite the evidence for IxE when tested with trait‐specific models, estimates of **I**_**post‐bird**_ and **I**_**post‐cichlid**_ were qualitatively very similar to that of **I**_**pre**_ (Table 2). Using difference matrices (**D**) to compare each pair of **I** matrices revealed no significant cross‐context differences in the (co)variance structures for among‐individual behavioural variation from **I**_**pre**_ to either **I**_**post‐bird**_ or **I**_**post‐fish**_ (all **D** matrix elements close to zero and non‐significant; Table 5a,b). The **D** matrix showing differences from **I**_**post‐bird**_ or **I**_**post‐fish**_ did reveal some significant changes (Table 5c): an increase in the among‐individual variance for area covered, a decrease in the among‐individual variance for duration in the exposed zone, and a decrease in the among‐individual correlation between the number of freezings and the duration in the exposed zone. The **D** matrix traces show no significant changes in total variance (across all traits) between **I** matrices (estimates and 95% confidence intervals: pre‐ to post‐bird, −0.092 (−0.529, 0.324); pre‐ to post‐fish, 0.004 (−0.387, 0.392); post‐bird to post‐fish, 0.097 (−0.372, 0.576)). On the basis of the lack of significant change in total among‐individual behavioural variance, and no significant changes in any elements of the pre‐ to post‐predator **I** matrices, we conclude that our multivariate approach shows little evidence of IxE.

**Table 5:** Difference (**D**) variance‐covariance matrices for comparisons of (a) **I**_**pre**_ to **I**_**post‐bird**_; (b) pre‐predator to post‐cichlid reveal; (c) post‐bird strike to post‐cichlid reveal. Differences in variances appear on the diagonals, and differences in covariances off‐diagonal; 95% confidence intervals are taken from differences across 5,000 bootstrapped replicate pairs for each **D** matrix. Bold values indicate elements where 95% confidence intervals do not span zero

	Area	Exposed	Freezings	Shelter	Tracklength
(a)					
Area	−0.10 (−0.20, 0.02)				
Exposed	0.01 (−0.09, 0.11)	0.13 (−0.04, 0.30)			
Freezings	0.06 (−0.03, 0.15)	0.05 (−0.09, 0.19)	−0.06 (−0.20, 0.08)		
Shelter	0.02 (−0.08, 0.11)	−0.06 (−0.20, 0.06)	0.01 (−0.11, 0.12)	0.00 (−0.13, 0.13)	
Tracklength	−0.05 (−0.14, 0.04)	0.02 (−0.08, 0.11)	0.05 (−0.04, 0.14)	0.01 (−0.08, 0.10)	−0.08 (−0.18, 0.03)
(b)					
Area	0.06 (−0.08, 0.21)				
Exposed	0.01 (−0.07, 0.10)	−0.09 (−0.18, 0.00)			
Freezings	0.06 (−0.05, 0.17)	−0.09 (−0.19, 0.01)	0.05 (−0.12, 0.23)		
Shelter	−0.03 (−0.14, 0.07)	0.04 (−0.04, 0.13)	−0.04 (−0.16, 0.08)	0.01 (−0.12, 0.14)	
Tracklength	0.03 (−0.09, 0.13)	0.06 (−0.01, 0.14)	0.03 (−0.08, 0.13)	0.01 (−0.09, 0.10)	−0.02 (−0.15, 0.10)
(c)					
Area	**0.16 (0.03, 0.30)**				
Exposed	0.00 (−0.10, 0.10)	−**0.22 (**−**0.39,** −**0.06)**			
Freezings	0.00 (−0.12, 0.11)	−**0.15 (**−**0.28,** −**0.02)**	0.10 (−0.08, 0.27)		
Shelter	−0.05 −0.17, 0.06)	0.10 (−0.03, 0.23)	−0.05 (−0.19, 0.09)	0.01 (−0.15, 0.16)	
Tracklength	0.08 (−0.03, 0.18)	0.05 (−0.04, 0.14)	−0.02 (−0.12, 0.09)	−0.01 (−0.11, 0.11)	0.05 (−0.06, 0.18)

Given the lack of significant differentiation between the three context‐specific **I** matrices, we elected to fit one additional multivariate model post hoc, pooling all data (with all fixed and random effects as described earlier) to estimate an averaged (across all contexts) covariance matrix **I**_**all**_ that we subjected to eigen decomposition. Although obfuscating any IxE present (as suggested by single‐trait models but not supported by multi‐trait analyses), this allowed us to utilise data from all of the stress contexts at once, and thus generate a more precise estimate of the among‐individual behavioural (co)variation structure first estimated above (as **I**_**pre**_) utilising solely the OFT stress context. That is, we estimated among‐individual behavioural (co)variances using up to 16 measurements of each behaviour per individual (8 × OFT, 4 × post‐bird strike, 4 × post‐cichlid reveal), and including fixed effects to control for environmental variables and—crucially—population‐level plasticity in each trait across contexts. Similar to **I**_**pre**_, eigen decomposition of **I**_**all**_ showed no clear support for the idea of a single major axis of among‐individual behavioural variation: together, the first two axes explained over 90% of the total among‐individual variation (EV1_all_ = 57.4%; EV2_all_ = 34.2%). Trait loadings were equivalent to **I**_pre_ (see Figure 2), and confidence intervals tightened around strongly loading traits (EV1_all_: exposed duration and number of freezings vs. shelter duration; EV2_all_: area covered and tracklength vs. number of freezings; Figure S2), lending support to statistical significance of the presence of “alternative strategies” of behavioural stress coping styles which are consistent across stress contexts.

## 4 DISCUSSION

We found significant repeatable among‐individual (co)variation (“personality”) in all behaviours, and within each stress context. We also found strong evidence for changes in mean behaviour (population‐level behavioural plasticity) due to the predator stimuli. At the among‐individual level, the majority of cross‐context correlations were significantly different from a “perfect” correlation, thus indicating the presence of individual‐by‐environment interactions (IxE). However, in contrast to the significant (albeit low) IxE found in these pairwise correlations, our multivariate analyses provided little evidence that individual variation in plasticity was causing instability of the **I** matrix across contexts. We found no evidence for changes in the structure of the among‐individual covariance matrix (**I**) between pre‐ and post‐predator contexts, and only minor changes between the two post‐predator contexts. We also found no cross‐context changes in the total among‐individual variation in measured behaviours. Our investigation of the **I** matrix revealed no single major axis of behavioural variation (and we found that **I**_pre_ was qualitatively similar to the overall **I** matrix, **I**_all_, having pooled across all contexts and stages). Rather than the simple “risk‐prone–risk‐averse” continuum as posited by the original coping styles model, our two axes indicate a more complex level of variation in individual strategies.

The strong evidence of behavioural change across different stress contexts that we found at the population level, with general shifts towards a “more shy” behavioural mean, was expected: behaviour is often highly flexible, enabling individuals to react quickly in response to environmental changes (Ghalambor, Angeloni, & Carroll, 2010; Komers, 1997). In the context of the stress literature, the adaptive response to stressors includes various processes (neuroendocrine, physiological and behavioural) that enable an individual to redirect behaviour and energy in order to establish homeostasis (Johnson, Kamilaris, Chrousos, & Gold, 1992). In this study, we found that our two moderate stress contexts induced similar amounts of population‐level change (relative to the mild stressor of the pre‐predator OFT) for several behaviours: the mean reduction in area covered, duration in the exposed zone, and distance travelled were equivalent in both bird strike and cichlid reveal.

One intriguing result is that the number of freezings increased significantly after the cichlid reveal, yet after the bird strike there was a marginally non‐significant decrease. Our expectation of a tendency towards “more shy” behaviours under greater stress had led us to predict an increase in the number of freezings in both post‐predator contexts. However, this result might best be explained by the change in another behavioural variable: the mean increase in duration in the shelter post‐bird strike was almost double that of post‐cichlid reveal. We note that there is a significant negative correlation between variation in shelter use and in the number of freezings at both the among‐ and within‐individual level for each stress context; also that the mean number of freezings per second out of the shelter increased across stages in both predator types (pre‐bird = 0.028 ± 0.002, post‐bird = 0.032 ± 0.002; pre‐cichlid = 0.030 ± 0.002, post‐cichlid = 0.040 ± 0.002). Taken together, these results provide a simpler explanation for the apparent increase in “bolder” freezing behaviour (i.e. a decrease in the number of freezings) under increased stress: guppies increased their shelter use far more post‐bird strike compared to post‐cichlid reveal, with the result that individuals had fewer opportunities for freezing behaviour post‐bird strike.

While population‐level plasticity informs us about the average change in behaviour within said population, plasticity is itself the property of an individual (or, more specifically, a genotype; Falconer & Mackay, 1996; Via & Lande, 1985). Individuals can vary in the extent of their plasticity across different environments or contexts, and this phenomenon is known variously as individual variation in plasticity, individual differences in slopes (when using reaction norms), and individual‐by‐environment interactions (IxE). All of these mean the same thing: that individuals (or genotypes) do not change their phenotype (in this case, their behaviour) at the same rate with respect to changes in their environment. For behaviours related to coping styles, IxE would suggest that individuals do not maintain their position along the putative “risk‐prone–risk‐averse” axis relative to others, and instead alter their relative performance as the environment (e.g. stressor severity) changes.

When testing each behavioural trait separately, we found evidence of statistically significant IxE across at least one pair of contexts for all five measured behaviours. Significant IxE was typically found between mild (pre‐predator OFT) and moderate (post‐predator) stress contexts: for all but duration in the exposed zone, the correlations across the two types of post‐predator contexts were not significantly different from +1 (i.e. where *r* = +1 means that individual performance is perfectly correlated such that there is an absence of IxE in terms of rank order changes). The existence of IxE from pre‐ to post‐predator contexts indicates some changes in the rank order of the relative performance of individuals across contexts, although all correlations were also significantly greater than zero—suggesting that relative performance is generally predictable across all contexts.

While we did find statistically significant IxE in our trait‐specific tests, our second approach to analysing IxE (via examination of the **I** matrix) suggests that multivariate personality structure was largely conserved across each of the stress contexts—particularly between mild and moderate—thus indicating an apparent lack of IxE at the multivariate level. How might we reconcile these seemingly conflicting results? Rather than the reaction norm models (typically formulated as random regression mixed models) that are often used for the study of individual plasticity variation, we employed “character state” models: the character state approach aids interpretation by estimating the among‐individual variance in each context and the covariation between them (see Figure 1 and associated legend). This contrasts with reaction norm models in which (co)variances in intercepts and slopes are estimated, but on different scales such that their absolute and relative magnitudes are less easily interpreted (see Brommer, 2013 for discussion). Here, our powerful study design enables us to detect statistically significant changes in variation and imperfect correlations across contexts in the univariate case, but our use of multivariate character state models better enables assessment of the magnitude of these changes. In this case, our univariate models demonstrate IxE effects that are statistically significant but small, ultimately producing only minor effects on the actual phenotypic values (and leading to the structure of the **I** matrix being largely conserved across stress contexts). As illustrated in Figure 4, the rank order changes tend to be relatively minor, such that relative performance is fairly well conserved across all contexts. We can therefore infer, for example, that an individual that covers a relatively large area (compared to its peers) in a mild stress context would also cover a relatively large area in a higher stress context, having taken into account the expectation that all fish are likely to cover less area overall in the higher stress context.

Here, we found that the structure of the **I** matrix between mild and moderate stress contexts was largely conserved; yet for the sake of interpretation, it may be fruitful to consider how larger IxE across contexts would have been manifest in **I**. We might have expected, for example, that increased stressor severity would increase the amount of among‐individual variation in behaviour (manifest as positive values on the diagonal of **D** matrices, and greater matrix traces in **I**_**post**_). This would have meant that, in addition to the changes in mean behaviour across stress contexts (population‐level behavioural plasticity), that individuals behave “more differently” from one another (which would be seen as a “fanning out” of the visualised reaction norms). Such a result would have been more consistent with the “two‐tier” model of stress coping styles described by Koolhaas et al. (2007), in which individuals differ not only in “coping style” (i.e. where their response lies on a putative risk‐prone—risk‐averse continuum) but also in their “responsiveness” (i.e. the magnitude of their response to the environmental stressor).

Here, not only did we find no difference in the amount of among‐individual variation across contexts, but the covariance structure of the **I** matrix also showed few significant differences in their elements (and none between the mild and moderate stress contexts). The relationships between traits are therefore neither decoupled nor more tightly integrated under higher levels of stress. Accepting this conservation of **I** across contexts, our eigen decomposition of the post hoc matrix estimate based on all data (across contexts) best enables us to scrutinise the major axes of among‐individual behavioural variation (see Houslay & Wilson, 2017 for further discussion of this approach). While the behavioural component of “coping styles” describes different ways in which individuals can attain successful environmental control (Koolhaas et al., 1999, 2007), the structure of **I** here does not really conform to expectations from verbal models in the literature. Specifically, behavioural coping style is typically portrayed as a single major axis of variation or even a simple bimodal distribution (although note that much of the work focusing on “alternative response patterns” is informed by studies using artificial selection lines, which may lead to oversimplification of the true nature of the underlying behavioural variation; Réale et al., 2010). As noted previously, while the “two‐tier” model does embrace the idea of greater complexity in among‐individual behavioural variation, it still implies the existence of a single axis denoting the type of behavioural response, while a second dimension shows variation in the magnitude of that response (Koolhaas, de Boer, Coppens, & Buwalda, 2010; Koolhaas et al., 2007).

In this study, rather than the single major axis posited by the verbal models of stress coping styles, we instead found two major axes of among‐individual variation in behaviour. The first axis loaded strongly on increased shelter duration in one direction, while all other traits loaded in the other direction, indicating variation on a continuum from high use of the shelter (shyer, risk‐averse individuals) to other behaviours (nominally bolder, more risk‐prone individuals). The second axis loaded heavily on increased number of freezings in one direction, and greater area covered and tracklength (i.e. distance travelled) in the other direction. Increased duration in the exposed zone also loaded (non‐significantly) in the same direction as the increased number of freezings, therefore indicating that increased area covered and distance travelled were not associated with time spent in the central exposed zone. Together, these two axes potentially correspond to multiple strategies for behavioural control of a stressful environment: individuals may seek refuge in the shelter, but otherwise may adopt a strategy of either freezing in place (typically in an exposed area) or actively trying to escape the situation. “Freezing” vs. “active startle” have been demonstrated previously as alternative stress‐response behaviours in guppies, using OFTs that did not include a shelter (Fischer, Schwartz, Hoke, & Soares, 2015). We note that freezing and hiding are both effectively passive, “conservation‐withdrawal” strategies, and might therefore be considered alternatives among more “reactive” individuals (Øverli et al., 2007). Our results do raise the question, however, of whether simple additions to the testing environment can reveal complex behavioural (co)variation that might otherwise go unnoticed.

Overall, our results provide behavioural evidence in support of the concept of coping styles, but also highlight that the full range of their underlying variation might not be readily captured analytically by a simple, single‐axis paradigm, even when considering behaviour alone. We have used this study to demonstrate how character state models—in comparison to a random regression approach—enable a better understanding of the magnitude of IxE and its consequences for among‐individual variance in observed traits, by directly estimating changes in variance across contexts as well as testing specific hypotheses regarding the cross‐context covariance. We also show that, even when behavioural flexibility enables populations (and individuals) to respond to environmental changes, personality structure can be strongly conserved. This stability of relative behaviour means that—while we do not know how selection on behavioural types might differ—the material upon which selection acts can show consistency across contexts.

## AUTHORS' CONTRIBUTIONS

M.V. and A.J.W. designed the experiment; T.M.H., M.V. and A.J.G. collected the data; T.M.H. analysed the data; T.M.H., A.J.Y. and A.J.W. led the writing of the manuscript. All authors contributed critically to manuscript drafts and gave final approval for publication.

## DATA ACCESSIBILITY

Data deposited in the Dryad Digital Repository https://doi.org/10.5061/dryad.g0m41, (Houslay, Vierbuchen, Grimmer, Young, & Wilson, 2017).
