# Supplementary material for: Testing the stability of behavioural coping style across stress contexts in the Trinidadian guppy
Source: Funct Ecol. 2017 Sep 24;32(2):424–38. doi: 10.1111/1365-2435.12981 (PMC5836853; doi:10.1111/1365-2435.12981)

Functional Ecology

Testing the stability of behavioural coping style across stress contexts in the Trinidadian guppy

Thomas M. Houslay, Maddalena Vierbuchen, Andrew J. Grimmer, Andrew J. Young and Alastair J. Wilson

Coping with challenging environments and situations is a necessary part of life. Previous research has shown that individuals within a population may vary in the way they respond to stress, and this variation is considered to exist on a simple spectrum of 'reactive' to 'proactive' (or 'risk-averse' to 'risk-prone') styles of coping. We wanted to test this idea of a single underlying axis of coping styles, using a suite of behaviours related to risk-taking. We also wanted to test individual consistency across different stress contexts: we expect individuals to adjust their behaviour in response to a stressor, but do they all change at the same rate, or is there variation in how they respond? Does such variation mean that 'coping styles' are not stable across contexts?

We measured these risk-taking behaviours in the Trinidadian *guppy (Poecilia reticulata)* repeatedly across multiple different 'stress contexts': one mild (an open field trial), and two moderate (simulated predator attacks, a 'bird strike' and the reveal of a predatory cichlid fish). Using multivariate modelling approaches, informed by recent advances in the fields of animal personality and individual plasticity variation, we assessed both the presence and cross-context stability of coping styles in this population.

Individuals did show repeatable differences in their behaviour in the mild stress context, supporting the general concept of coping styles. As expected, we saw strong changes in 'average behaviour' in response to changes in stress context, yet limited evidence of variation among individuals in the magnitude of their response, suggesting that coping styles are also consistent across contexts in this species. However, our investigation of relationships between these behaviours at the among-individual level revealed no single major axis of variation: rather than the simple 'risk-prone—risk-averse' continuum as posited by the original coping styles model, our results indicate a more complex level of variation in individual strategies.

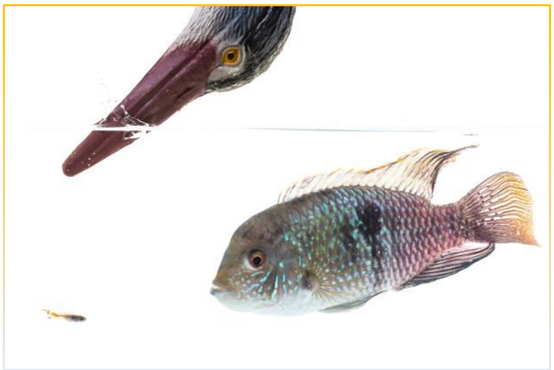

Supplement: Supplementary file 1 [file FEC-32-424-s001.pdf]
