# Supplementary material for: Testing the stability of behavioural coping style across stress contexts in the Trinidadian guppy
Source: Funct Ecol. 2017 Sep 24;32(2):424–38. doi: 10.1111/1365-2435.12981 (PMC5836853; doi:10.1111/1365-2435.12981)
Supplement: Supplementary file 2 [file FEC-32-424-s002.pdf]

Title: Testing the stability of behavioural coping style across stress contexts in the Trinidadian guppy

Running headline: Cross-context stability of coping styles

Key words: Coping styles, animal personality, individual plasticity, individual by environment interactions, behavioural syndromes, multi-response model, individual differences, *Poecilia reticulata.*

Thomas M. Houslay*[1], Maddalena Vierbuchen[1], Andrew J. Grimmer[1,2], Andrew J. Young[1], Alastair J. Wilson[1]

[1] Centre for Ecology and Conservation, University of Exeter, Penryn, Cornwall, TR10 9FE, UK.

[2] School of Biological & Marine Sciences, Plymouth University, Devon, PL4 8AA, UK.

*Corresponding author: t.houslay@exeter.ac.uk

Supporting Information

Contents

Figure S1: Tank configurations for (a) bird strike, and (b) cichlid reveal assays.

Figure S2: Trait loadings on the first two eigenvectors from $\mathbf{I}_{all}$ (the **I** matrix for behavioural variation pooled across all contexts and assay types).

Table S1: Conditional Wald *F*-tests for fixed effects in multivariate mixed-effects models.

Table S2: Fixed effects summaries from multivariate mixed-effects models.

Table S3: Effects of 'stage' (pre- to post-) in the control group, where no predator stimulus was applied.

Appendix S1: Annotated R code for multivariate mixed effects-models and the parametric bootstrapping procedure used.

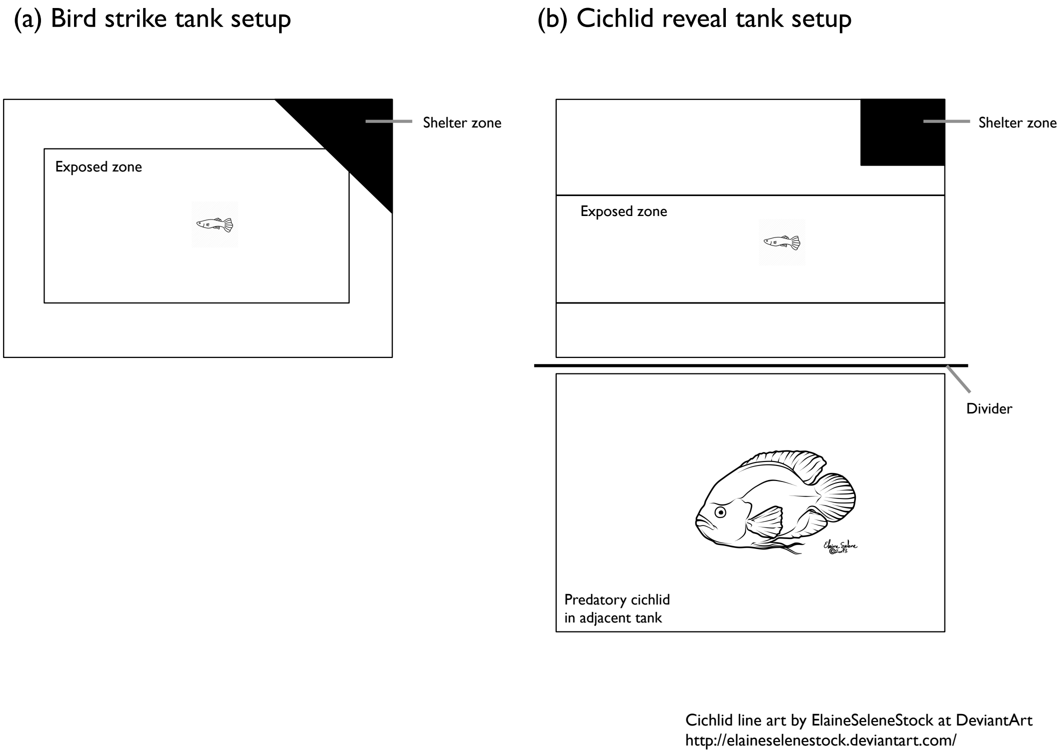

Figure S1: Tank configurations for (a) bird strike, and (b) cichlid reveal assays.

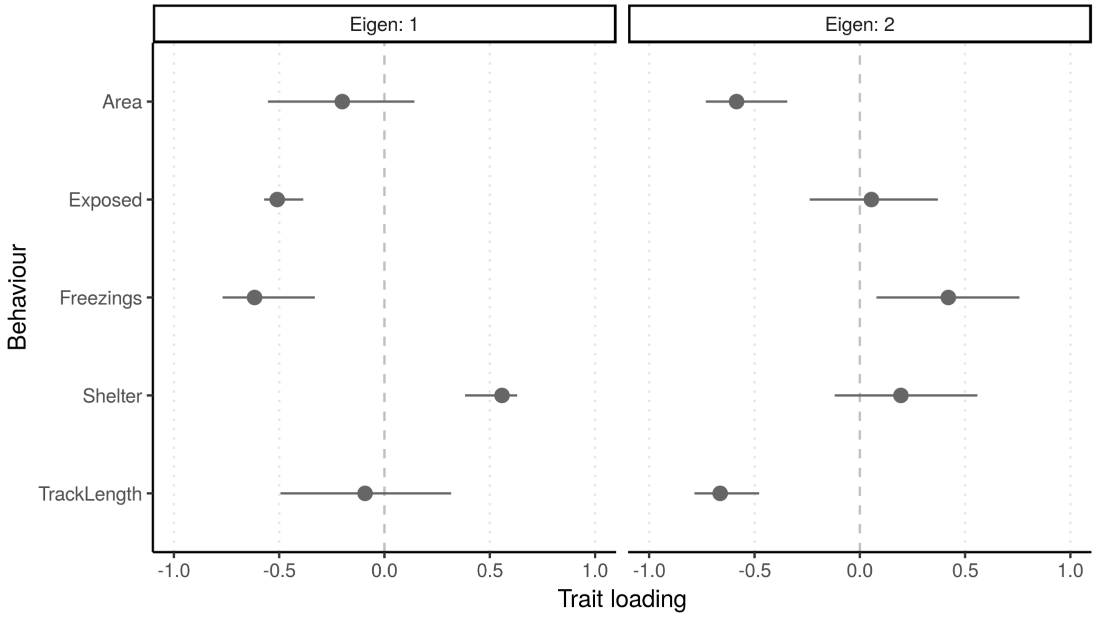

Figure S2: Trait loadings on the first two eigenvectors from $\mathbf{I}_{all}$ (the **I** matrix for behavioural variation pooled across all contexts and assay types).

Table S1: Conditional Wald *F*-tests for fixed effects in multivariate mixed-effects models.

Context	Parameter	df	F	P
Pooled pre	trait	5,82.3	4910	<0.001
	trait:Assay	5,703.5	60.3	<0.001
	trait:Sex (Male)	5,97.1	11.9	<0.001
	trait:Replicate	5,715.0	7.5	<0.001
	trait:Order	5,742.8	2.5	0.03
	trait:Time	5,778.4	0.8	0.52
	trait:Block	10,179.5	7.8	<0.001
	trait:Tank	70,263.8	1.1	0.25
Post-bird	trait	5,82.4	3039	<0.001
	trait:Sex (Male)	5,122.8	10.2	<0.001
	trait:Replicate	5,304.5	4.5	<0.001
	trait:Order	5,378.4	1.8	0.11
	trait:Time	5,351.1	0.08	0.99
	trait:Block	10,189.8	2.4	0.01
	trait:Tank	70,276.5	0.91	0.68
Post-fish	trait	5,81.8	925.7	<0.001
	trait:Sex (Male)	5,88.9	11.8	<0.001
	trait:Replicate	5,303.4	7.9	<0.001
	trait:Order	5,365.3	1.9	0.09
	trait:Time	5,373.0	1.5	0.2
	trait:Block	10,167.0	6.6	<0.001
	trait:Tank	70,267.5	1.1	0.28

Table S2: Fixed effects estimates, standard errors and z-ratios from multivariate mixed models for observations at: (a) pooled pre-stimulus; (b) post-bird strike; (c) post-cichlid reveal; (d) all stages and configurations. These values are taken from models corresponding to 1D, 2D, 3D, and the full unstructured model.

(a)

	solution	std error	z ratio
trait_Area:Block_Tank_GRIM_1_Q10b	0	NA	NA
trait_Area:Block_Tank_GRIM_1_Q11a	-0.261291754	0.275812438	-0.94735305
trait_Area:Block_Tank_GRIM_1_Q12b	-0.594794205	0.283453371	-2.098384655
trait_Area:Block_Tank_GRIM_1_Q9a	-0.035571016	0.284646973	-0.124965376
trait_Area:Block_Tank_GRIM_2_R10a	0	NA	NA
trait_Area:Block_Tank_GRIM_2_R11a	-0.022549669	0.296814403	-0.075972287
trait_Area:Block_Tank_GRIM_2_R12a	-0.197067157	0.309401073	-0.63693107
trait_Area:Block_Tank_GRIM_2_R9a	0.576791343	0.311745817	1.850197537
trait_Area:Block_Tank_SMV_1	0	NA	NA
trait_Area:Block_Tank_SMV_2	0.370487114	0.319778461	1.158574322
trait_Area:Block_Tank_SMV_3	0.387965377	0.360392976	1.07650649
trait_Area:Block_Tank_SMV_4	0.129653696	0.355944521	0.364252539
trait_Area:Block_Tank_SMV_5	0.26405794	0.332018204	0.795311633
trait_Area:Block_Tank_SMV_6	0.117834258	0.360264016	0.327077512
trait_Area:Block_Tank_TMH_P10a	0	NA	NA
trait_Area:Block_Tank_TMH_P10b	0.068429208	0.317754286	0.215352589
trait_Area:Block_Tank_TMH_P9a	0.083906114	0.332463853	0.252376653
trait_Area:Block_Tank_TMH_P9b	0.109357246	0.307701194	0.355400785
trait_Exposed:Block_Tank_GRIM_1_Q10b	0	NA	NA
trait_Exposed:Block_Tank_GRIM_1_Q11a	-0.267134621	0.259654703	-1.028807172
trait_Exposed:Block_Tank_GRIM_1_Q12b	-0.733354927	0.266725397	-2.74947544
trait_Exposed:Block_Tank_GRIM_1_Q9a	-0.307379293	0.267963115	-1.147095536
trait_Exposed:Block_Tank_GRIM_2_R10a	0	NA	NA
trait_Exposed:Block_Tank_GRIM_2_R11a	0.695118118	0.27944738	2.487474091
trait_Exposed:Block_Tank_GRIM_2_R12a	0.089780452	0.291349139	0.308154172
trait_Exposed:Block_Tank_GRIM_2_R9a	0.546524321	0.293792779	1.860237418
trait_Exposed:Block_Tank_SMV_1	0	NA	NA
trait_Exposed:Block_Tank_SMV_2	-0.1227487	0.300876141	-0.407970866
trait_Exposed:Block_Tank_SMV_3	-0.363576803	0.3395619	-1.070723198
trait_Exposed:Block_Tank_SMV_4	0.054766928	0.335000276	0.163483231
trait_Exposed:Block_Tank_SMV_5	-0.146656695	0.312199984	-0.469752409
trait_Exposed:Block_Tank_SMV_6	0.082006204	0.339452509	0.241583731
trait_Exposed:Block_Tank_TMH_P10a	0	NA	NA
trait_Exposed:Block_Tank_TMH_P10b	-0.205667701	0.298779634	-0.688359169
trait_Exposed:Block_Tank_TMH_P9a	-0.129831421	0.312675405	-0.415227481
trait_Exposed:Block_Tank_TMH_P9b	0.137628566	0.28937012	0.475614297
trait_Freezings:Block_Tank_GRIM_1_Q10b	0	NA	NA
trait_Freezings:Block_Tank_GRIM_1_Q11a	-0.025380374	0.294782562	-0.086098628

trait_Freezings:Block_Tank_GRIM_1_Q12b	-0.56535985	0.303296964	-1.864047179
trait_Freezings:Block_Tank_GRIM_1_Q9a	-0.229269629	0.304276399	-0.753491329
trait_Freezings:Block_Tank_GRIM_2_R10a	0	NA	NA
trait_Freezings:Block_Tank_GRIM_2_R11a	0.318249257	0.317148827	1.003469759
trait_Freezings:Block_Tank_GRIM_2_R12a	-0.028986392	0.330265882	-0.087766837
trait_Freezings:Block_Tank_GRIM_2_R9a	-0.147905986	0.332191333	-0.445243363
trait_Freezings:Block_Tank_SMV_1	0	NA	NA
trait_Freezings:Block_Tank_SMV_2	-0.399253036	0.342487827	-1.165743727
trait_Freezings:Block_Tank_SMV_3	-0.493097965	0.384458168	-1.282578981
trait_Freezings:Block_Tank_SMV_4	-0.149904439	0.380803154	-0.393653354
trait_Freezings:Block_Tank_SMV_5	-0.186599038	0.356096916	-0.524011947
trait_Freezings:Block_Tank_SMV_6	0.372285223	0.384347401	0.96861647
trait_Freezings:Block_Tank_TMH_P10a	0	NA	NA
trait_Freezings:Block_Tank_TMH_P10b	-0.002047864	0.340831063	-0.006008443
trait_Freezings:Block_Tank_TMH_P9a	-0.065253938	0.35646069	-0.183060683
trait_Freezings:Block_Tank_TMH_P9b	0.259503843	0.329936699	0.786526154
trait_Shelter:Block_Tank_GRIM_1_Q10b	0	NA	NA
trait_Shelter:Block_Tank_GRIM_1_Q11a	0.316695378	0.237519838	1.333342852
trait_Shelter:Block_Tank_GRIM_1_Q12b	0.919750265	0.244376931	3.763654204
trait_Shelter:Block_Tank_GRIM_1_Q9a	0.166302743	0.245166393	0.678326018
trait_Shelter:Block_Tank_GRIM_2_R10a	0	NA	NA
trait_Shelter:Block_Tank_GRIM_2_R11a	-0.334468549	0.255544041	-1.308848945
trait_Shelter:Block_Tank_GRIM_2_R12a	-0.095069569	0.2661341	-0.357224305
trait_Shelter:Block_Tank_GRIM_2_R9a	-0.339406957	0.267682304	-1.267946933
trait_Shelter:Block_Tank_SMV_1	0	NA	NA
trait_Shelter:Block_Tank_SMV_2	-0.051756963	0.275928805	-0.187573613
trait_Shelter:Block_Tank_SMV_3	-0.091788544	0.309774828	-0.296307302
trait_Shelter:Block_Tank_SMV_4	0.245114563	0.306815841	0.798898003
trait_Shelter:Block_Tank_SMV_5	-0.018855119	0.286892578	-0.065721878
trait_Shelter:Block_Tank_SMV_6	0.260264511	0.309679358	0.84043222
trait_Shelter:Block_Tank_TMH_P10a	0	NA	NA
trait_Shelter:Block_Tank_TMH_P10b	0.187780846	0.274592229	0.68385346
trait_Shelter:Block_Tank_TMH_P9a	0.074925274	0.287182471	0.260897795
trait_Shelter:Block_Tank_TMH_P9b	-0.048616536	0.265815134	-0.182896041
trait_TrackLen:Block_Tank_GRIM_1_Q10b	0	NA	NA
trait_TrackLen:Block_Tank_GRIM_1_Q11a	-0.367429957	0.268844533	-1.366700497
trait_TrackLen:Block_Tank_GRIM_1_Q12b	-0.455054586	0.276616287	-1.645075171
trait_TrackLen:Block_Tank_GRIM_1_Q9a	-0.023609471	0.277500394	-0.085079055
trait_TrackLen:Block_Tank_GRIM_2_R10a	0	NA	NA
trait_TrackLen:Block_Tank_GRIM_2_R11a	-0.13189995	0.289243929	-0.456016312
trait_TrackLen:Block_Tank_GRIM_2_R12a	-0.246638879	0.301225372	-0.818785209
trait_TrackLen:Block_Tank_GRIM_2_R9a	0.20861597	0.302958134	0.688596696
trait_TrackLen:Block_Tank_SMV_1	0	NA	NA
trait_TrackLen:Block_Tank_SMV_2	0.541257548	0.312334263	1.732943236
trait_TrackLen:Block_Tank_SMV_3	0.752068447	0.350605139	2.14505825
trait_TrackLen:Block_Tank_SMV_4	-0.009159756	0.347287716	-0.026375123

trait_TrackLen:Block_Tank_SMV_5	0.264745161	0.324760487	0.815201269
trait_TrackLen:Block_Tank_SMV_6	-0.258557958	0.350496296	-0.737690985
trait_TrackLen:Block_Tank_TMH_P10a	0	NA	NA
trait_TrackLen:Block_Tank_TMH_P10b	-0.142886785	0.310837278	-0.459683556
trait_TrackLen:Block_Tank_TMH_P9a	0.088554459	0.32508415	0.272404727
trait_TrackLen:Block_Tank_TMH_P9b	-0.258208438	0.300898083	-0.858125898
trait_Area:Block_GRIM_1	0	NA	NA
trait_Area:Block_GRIM_2	-0.26086403	0.298540483	-0.873797841
trait_Area:Block_SMV	0	NA	NA
trait_Area:Block_TMH	0.441793878	0.349615483	1.263656501
trait_Exposed:Block_GRIM_1	0	NA	NA
trait_Exposed:Block_GRIM_2	-0.40513937	0.281144113	-1.441038069
trait_Exposed:Block_SMV	0	NA	NA
trait_Exposed:Block_TMH	-0.486000142	0.330709111	-1.46956986
trait_Freezings:Block_GRIM_1	0	NA	NA
trait_Freezings:Block_GRIM_2	0.026676874	0.318575939	0.083737881
trait_Freezings:Block_SMV	0	NA	NA
trait_Freezings:Block_TMH	-1.038667021	0.370560695	-2.802960582
trait_Shelter:Block_GRIM_1	0	NA	NA
trait_Shelter:Block_GRIM_2	0.114245573	0.256718281	0.445023128
trait_Shelter:Block_SMV	0	NA	NA
trait_Shelter:Block_TMH	-0.445004027	0.298474842	-1.490926417
trait_TrackLen:Block_GRIM_1	0	NA	NA
trait_TrackLen:Block_GRIM_2	0.005383185	0.290565975	0.018526549
trait_TrackLen:Block_SMV	0	NA	NA
trait_TrackLen:Block_TMH	1.399961366	0.337710862	4.145443704
trait_Area:scale(preTimeNum)	-0.030408546	0.051904549	-0.585855127
trait_Exposed:scale(preTimeNum)	0.000942463	0.051686102	0.018234363
trait_Freezings:scale(preTimeNum)	0.014968137	0.048586917	0.308069286
trait_Shelter:scale(preTimeNum)	0.063562343	0.039027172	1.628668958
trait_TrackLen:scale(preTimeNum)	-0.072099017	0.04390274	-1.642244133
trait_Area:scale(Order, scale = FALSE)	-0.017683917	0.016537342	-1.069332454
trait_Exposed:scale(Order, scale = FALSE)	-0.049057679	0.016189711	-3.030176273
trait_Freezings:scale(Order, scale = FALSE)	-0.028913464	0.015500936	-1.865272146
trait_Shelter:scale(Order, scale = FALSE)	0.007047184	0.012537977	0.562067092
trait_TrackLen:scale(Order, scale = FALSE)	0.015822409	0.014129299	1.119829768
trait_Area:scale(Replicate, scale = FALSE)	0.016234624	0.027564572	0.588967029
trait_Exposed:scale(Replicate, scale = FALSE)	0.12392161	0.027420989	4.519224587
trait_Freezings:scale(Replicate, scale = FALSE)	0.104656808	0.025805816	4.05555116
trait_Shelter:scale(Replicate, scale = FALSE)	-0.041152008	0.020737065	-1.984466409
trait_TrackLen:scale(Replicate, scale = FALSE)	-0.073747902	0.023330253	-3.161041632
trait_Area:SexM	-0.594997131	0.323562187	-1.838895751
trait_Exposed:SexM	0.437992922	0.305878031	1.431920168
trait_Freezings:SexM	0.494136448	0.342989529	1.440675025
trait_Shelter:SexM	-0.007941277	0.276313573	-0.028740089
trait_TrackLen:SexM	-0.613189234	0.312650072	-1.96126369

trait_Area:Assay_Bird	0	NA	NA
trait_Area:Assay_Fish	0.129535488	0.060525499	2.140180415
trait_Exposed:Assay_Bird	0	NA	NA
trait_Exposed:Assay_Fish	-0.509592218	0.060287881	-8.45264765
trait_Freezings:Assay_Bird	0	NA	NA
trait_Freezings:Assay_Fish	0.142913633	0.056656207	2.522470892
trait_Shelter:Assay_Bird	0	NA	NA
trait_Shelter:Assay_Fish	-0.405937012	0.045503333	-8.921039053
trait_TrackLen:Assay_Bird	0	NA	NA
trait_TrackLen:Assay_Fish	0.350720464	0.051186412	6.851827481
trait_Area	2.052341031	0.20523178	10.00011321
trait_Exposed	1.459497782	0.193547908	7.540757225
trait_Freezings	0.792955002	0.218413891	3.630515438
trait_Shelter	0.764275323	0.175992156	4.342666962
trait_TrackLen	1.596969926	0.19917387	8.017969062

(b)

	solution	std error	z ratio
trait_Area:Block_Tank_GRIM_1_Q10b	0	NA	NA
trait_Area:Block_Tank_GRIM_1_Q11a	-0.558058828	0.260180825	-2.144888379
trait_Area:Block_Tank_GRIM_1_Q12b	-0.520926375	0.254139382	-2.049766433
trait_Area:Block_Tank_GRIM_1_Q9a	-0.019016556	0.265584174	-0.071602744
trait_Area:Block_Tank_GRIM_2_R10a	0	NA	NA
trait_Area:Block_Tank_GRIM_2_R11a	-0.043537765	0.270638231	-0.160870714
trait_Area:Block_Tank_GRIM_2_R12a	-0.289622216	0.278457264	-1.040095747
trait_Area:Block_Tank_GRIM_2_R9a	0.337722555	0.286987983	1.176782912
trait_Area:Block_Tank_SMV_1	0	NA	NA
trait_Area:Block_Tank_SMV_2	-0.018882291	0.293153496	-0.064410936
trait_Area:Block_Tank_SMV_3	0.018563459	0.332035224	0.055908101
trait_Area:Block_Tank_SMV_4	-0.272375187	0.32411146	-0.840375058
trait_Area:Block_Tank_SMV_5	-0.124640108	0.298249309	-0.417905774
trait_Area:Block_Tank_SMV_6	-0.35152807	0.331333105	-1.06095064
trait_Area:Block_Tank_TMH_P10a	0	NA	NA
trait_Area:Block_Tank_TMH_P10b	-0.437657745	0.287342592	-1.52312173
trait_Area:Block_Tank_TMH_P9a	-0.169425063	0.303241478	-0.558713352
trait_Area:Block_Tank_TMH_P9b	-0.043696247	0.278374483	-0.156969298
trait_Exposed:Block_Tank_GRIM_1_Q10b	0	NA	NA
trait_Exposed:Block_Tank_GRIM_1_Q11a	-0.400328427	0.381292659	-1.049924297
trait_Exposed:Block_Tank_GRIM_1_Q12b	-0.703547771	0.378618719	-1.858195952
trait_Exposed:Block_Tank_GRIM_1_Q9a	-0.307996461	0.390511088	-0.788700937
trait_Exposed:Block_Tank_GRIM_2_R10a	0	NA	NA
trait_Exposed:Block_Tank_GRIM_2_R11a	0.668400586	0.400431721	1.669199895
trait_Exposed:Block_Tank_GRIM_2_R12a	-0.009514311	0.413136734	-0.023029449
trait_Exposed:Block_Tank_GRIM_2_R9a	0.36093216	0.422082662	0.855121976
trait_Exposed:Block_Tank_SMV_1	0	NA	NA

trait_Exposed:Block_Tank_SMV_2	-0.332002179	0.434336984	-0.764388461
trait_Exposed:Block_Tank_SMV_3	-0.411169412	0.489308025	-0.840307926
trait_Exposed:Block_Tank_SMV_4	-0.103232596	0.480513391	-0.214838124
trait_Exposed:Block_Tank_SMV_5	-0.001460196	0.445397518	-0.00327841
trait_Exposed:Block_Tank_SMV_6	0.314957962	0.488592098	0.644623528
trait_Exposed:Block_Tank_TMH_P10a	0	NA	NA
trait_Exposed:Block_Tank_TMH_P10b	-0.250786673	0.428215265	-0.585655611
trait_Exposed:Block_Tank_TMH_P9a	-0.074083087	0.450480116	-0.164453622
trait_Exposed:Block_Tank_TMH_P9b	0.471974404	0.414625478	1.138315006
trait_Freezings:Block_Tank_GRIM_1_Q10b	0	NA	NA
trait_Freezings:Block_Tank_GRIM_1_Q11a	-0.24909158	0.321224782	-0.775443222
trait_Freezings:Block_Tank_GRIM_1_Q12b	-0.537759046	0.317287919	-1.694861399
trait_Freezings:Block_Tank_GRIM_1_Q9a	-0.127560379	0.328637187	-0.388149559
trait_Freezings:Block_Tank_GRIM_2_R10a	0	NA	NA
trait_Freezings:Block_Tank_GRIM_2_R11a	0.438291927	0.336303463	1.303263201
trait_Freezings:Block_Tank_GRIM_2_R12a	0.04387408	0.346642385	0.12656871
trait_Freezings:Block_Tank_GRIM_2_R9a	0.176421885	0.355128945	0.496782611
trait_Freezings:Block_Tank_SMV_1	0	NA	NA
trait_Freezings:Block_Tank_SMV_2	-0.391205836	0.364630277	-1.072883576
trait_Freezings:Block_Tank_SMV_3	-0.413039416	0.411322766	-1.004173485
trait_Freezings:Block_Tank_SMV_4	-0.110772457	0.403310694	-0.274657871
trait_Freezings:Block_Tank_SMV_5	0.30277586	0.372989031	0.811755401
trait_Freezings:Block_Tank_SMV_6	0.111092874	0.410626513	0.270544816
trait_Freezings:Block_Tank_TMH_P10a	0	NA	NA
trait_Freezings:Block_Tank_TMH_P10b	-0.233656532	0.358856796	-0.651113577
trait_Freezings:Block_Tank_TMH_P9a	-0.28442918	0.377897086	-0.752663067
trait_Freezings:Block_Tank_TMH_P9b	0.238853734	0.347524545	0.687300327
trait_Shelter:Block_Tank_GRIM_1_Q10b	0	NA	NA
trait_Shelter:Block_Tank_GRIM_1_Q11a	0.743798752	0.326979519	2.274756395
trait_Shelter:Block_Tank_GRIM_1_Q12b	1.080357881	0.321029329	3.365293402
trait_Shelter:Block_Tank_GRIM_1_Q9a	0.219316284	0.334115606	0.65640838
trait_Shelter:Block_Tank_GRIM_2_R10a	0	NA	NA
trait_Shelter:Block_Tank_GRIM_2_R11a	-0.339039105	0.341130167	-0.993870195
trait_Shelter:Block_Tank_GRIM_2_R12a	-0.203667779	0.351268533	-0.579806501
trait_Shelter:Block_Tank_GRIM_2_R9a	-0.449366345	0.361029645	-1.244679906
trait_Shelter:Block_Tank_SMV_1	0	NA	NA
trait_Shelter:Block_Tank_SMV_2	0.486925531	0.369676135	1.317167881
trait_Shelter:Block_Tank_SMV_3	0.410938895	0.417877579	0.983395415
trait_Shelter:Block_Tank_SMV_4	0.401935664	0.408796138	0.98321786
trait_Shelter:Block_Tank_SMV_5	-0.038484692	0.377051566	-0.102067451
trait_Shelter:Block_Tank_SMV_6	0.337390648	0.417072137	0.808950343
trait_Shelter:Block_Tank_TMH_P10a	0	NA	NA
trait_Shelter:Block_Tank_TMH_P10b	0.54889126	0.363038521	1.51193669
trait_Shelter:Block_Tank_TMH_P9a	0.454010252	0.382744581	1.186196421
trait_Shelter:Block_Tank_TMH_P9b	-0.029622783	0.351644988	-0.084240596
trait_TrackLen:Block_Tank_GRIM_1_Q10b	0	NA	NA

trait_TrackLen:Block_Tank_GRIM_1_Q11a	-0.619883377	0.258790557	-2.395309098
trait_TrackLen:Block_Tank_GRIM_1_Q12b	-0.515497835	0.255974855	-2.013861229
trait_TrackLen:Block_Tank_GRIM_1_Q9a	-0.078907167	0.264836074	-0.297947202
trait_TrackLen:Block_Tank_GRIM_2_R10a	0	NA	NA
trait_TrackLen:Block_Tank_GRIM_2_R11a	-0.094187579	0.271160718	-0.347349644
trait_TrackLen:Block_Tank_GRIM_2_R12a	-0.048752248	0.279577763	-0.17437813
trait_TrackLen:Block_Tank_GRIM_2_R9a	0.274697009	0.286222073	0.959733839
trait_TrackLen:Block_Tank_SMV_1	0	NA	NA
trait_TrackLen:Block_Tank_SMV_2	-0.069336569	0.29402745	-0.235816654
trait_TrackLen:Block_Tank_SMV_3	-0.016895188	0.331632198	-0.050945561
trait_TrackLen:Block_Tank_SMV_4	-0.271561486	0.325236956	-0.834965033
trait_TrackLen:Block_Tank_SMV_5	-0.103863684	0.300958121	-0.345110088
trait_TrackLen:Block_Tank_SMV_6	-0.41105915	0.331094201	-1.241517215
trait_TrackLen:Block_Tank_TMH_P10a	0	NA	NA
trait_TrackLen:Block_Tank_TMH_P10b	-0.481010271	0.289492596	-1.661563293
trait_TrackLen:Block_Tank_TMH_P9a	-0.43916702	0.30477107	-1.440973449
trait_TrackLen:Block_Tank_TMH_P9b	-0.286588544	0.280340241	-1.022288283
trait_Area:Block_GRIM_1	0	NA	NA
trait_Area:Block_GRIM_2	-0.283839133	0.268566105	-1.056868783
trait_Area:Block_SMV	0	NA	NA
trait_Area:Block_TMH	0.608358743	0.360038478	1.689704797
trait_Exposed:Block_GRIM_1	0	NA	NA
trait_Exposed:Block_GRIM_2	-0.262895283	0.398398049	-0.659880951
trait_Exposed:Block_SMV	0	NA	NA
trait_Exposed:Block_TMH	-0.026904142	0.510843701	-0.052666093
trait_Freezings:Block_GRIM_1	0	NA	NA
trait_Freezings:Block_GRIM_2	-0.065391413	0.334278499	-0.195619559
trait_Freezings:Block_SMV	0	NA	NA
trait_Freezings:Block_TMH	-0.062705328	0.435036914	-0.144137948
trait_Shelter:Block_GRIM_1	0	NA	NA
trait_Shelter:Block_GRIM_2	0.467341257	0.33876278	1.379553143
trait_Shelter:Block_SMV	0	NA	NA
trait_Shelter:Block_TMH	-0.777593933	0.448101698	-1.735306821
trait_TrackLen:Block_GRIM_1	0	NA	NA
trait_TrackLen:Block_GRIM_2	-0.317189993	0.26961137	-1.176471129
trait_TrackLen:Block_SMV	0	NA	NA
trait_TrackLen:Block_TMH	1.139021275	0.349492683	3.259070452
trait_Area:scale(preTimeNum)	0.01535016	0.107023724	0.143427642
trait_Exposed:scale(preTimeNum)	0.001692853	0.132789025	0.012748438
trait_Freezings:scale(preTimeNum)	-0.009086295	0.118830619	-0.076464254
trait_Shelter:scale(preTimeNum)	0.001832597	0.128490489	0.014262511
trait_TrackLen:scale(preTimeNum)	-0.022357865	0.094302624	-0.237086348
trait_Area:scale(Order, scale = FALSE)	0.005736583	0.020111297	0.285241826
trait_Exposed:scale(Order, scale = FALSE)	-0.046456438	0.025465755	-1.82427099
trait_Freezings:scale(Order, scale = FALSE)	-0.011155984	0.0224677	-0.496534316
trait_Shelter:scale(Order, scale = FALSE)	0.016620899	0.024165841	0.68778483

trait_TrackLen:scale(Order, scale = FALSE)	0.014926214	0.017948563	0.8316105
trait_Area:scale(Replicate, scale = FALSE)	-0.029996607	0.033191212	-0.903751508
trait_Exposed:scale(Replicate, scale = FALSE)	0.038839129	0.041174999	0.943269708
trait_Freezings:scale(Replicate, scale = FALSE)	0.12969127	0.036855107	3.518949806
trait_Shelter:scale(Replicate, scale = FALSE)	-0.069288669	0.039851624	-1.738666149
trait_TrackLen:scale(Replicate, scale = FALSE)	-0.047295152	0.02924357	-1.61728377
trait_Area:SexM	0.020535053	0.347652764	0.059067712
trait_Exposed:SexM	0.380983184	0.488676438	0.779622578
trait_Freezings:SexM	0.284126434	0.417453952	0.680617426
trait_Shelter:SexM	-0.142755581	0.431474513	-0.330855187
trait_TrackLen:SexM	-0.19167768	0.335115192	-0.571975501
trait_Area	1.175443146	0.194025363	6.058193243
trait_Exposed	0.86995043	0.282751832	3.076727824
trait_Freezings	0.638552221	0.238623021	2.6759875
trait_Shelter	1.266464511	0.243403128	5.203156266
trait_TrackLen	1.052989007	0.192164941	5.479610384

(c)

	solution	std error	z ratio
trait_Area:Block_Tank_GRIM_1_Q10b	0	NA	NA
trait_Area:Block_Tank_GRIM_1_Q11a	-0.382994011	0.326343262	-1.173592516
trait_Area:Block_Tank_GRIM_1_Q12b	-0.489551559	0.337161283	-1.451980355
trait_Area:Block_Tank_GRIM_1_Q9a	0.108506337	0.336857696	0.322113279
trait_Area:Block_Tank_GRIM_2_R10a	0	NA	NA
trait_Area:Block_Tank_GRIM_2_R11a	-0.209452297	0.352587403	-0.59404362
trait_Area:Block_Tank_GRIM_2_R12a	-0.144365097	0.369848112	-0.390336173
trait_Area:Block_Tank_GRIM_2_R9a	0.712791471	0.372406963	1.914012199
trait_Area:Block_Tank_SMV_1	0	NA	NA
trait_Area:Block_Tank_SMV_2	-0.25138173	0.384531689	-0.653734755
trait_Area:Block_Tank_SMV_3	0.2977268	0.425998747	0.698891259
trait_Area:Block_Tank_SMV_4	-0.442395875	0.429109202	-1.030963385
trait_Area:Block_Tank_SMV_5	0.116759305	0.392164241	0.297730627
trait_Area:Block_Tank_SMV_6	0.116216965	0.425058648	0.273413952
trait_Area:Block_Tank_TMH_P10a	0	NA	NA
trait_Area:Block_Tank_TMH_P10b	-0.351563228	0.375513814	-0.936219161
trait_Area:Block_Tank_TMH_P9a	-0.163707173	0.39236372	-0.417233207
trait_Area:Block_Tank_TMH_P9b	-0.139765755	0.363667944	-0.384322449
trait_Exposed:Block_Tank_GRIM_1_Q10b	0	NA	NA
trait_Exposed:Block_Tank_GRIM_1_Q11a	-0.290884357	0.21410366	-1.358614592
trait_Exposed:Block_Tank_GRIM_1_Q12b	-0.575470682	0.221395358	-2.599289735
trait_Exposed:Block_Tank_GRIM_1_Q9a	-0.18307461	0.220964048	-0.828526683
trait_Exposed:Block_Tank_GRIM_2_R10a	0	NA	NA
trait_Exposed:Block_Tank_GRIM_2_R11a	-0.083556607	0.231935917	-0.360257299
trait_Exposed:Block_Tank_GRIM_2_R12a	0.275319936	0.244492694	1.126086557
trait_Exposed:Block_Tank_GRIM_2_R9a	0.316981967	0.247247188	1.28204478

trait_Exposed:Block_Tank_SMV_1	0	NA	NA
trait_Exposed:Block_Tank_SMV_2	-0.176314391	0.25412805	-0.69380138
trait_Exposed:Block_Tank_SMV_3	-0.103625951	0.280621472	-0.369273065
trait_Exposed:Block_Tank_SMV_4	-0.280369085	0.284309674	-0.986139802
trait_Exposed:Block_Tank_SMV_5	-0.035892086	0.255169385	-0.140659846
trait_Exposed:Block_Tank_SMV_6	0.123160661	0.280069593	0.439750206
trait_Exposed:Block_Tank_TMH_P10a	0	NA	NA
trait_Exposed:Block_Tank_TMH_P10b	0.232107641	0.244351884	0.949890941
trait_Exposed:Block_Tank_TMH_P9a	0.148247919	0.255385998	0.580485698
trait_Exposed:Block_Tank_TMH_P9b	-0.101369614	0.236843717	-0.428002123
trait_Freezings:Block_Tank_GRIM_1_Q10b	0	NA	NA
trait_Freezings:Block_Tank_GRIM_1_Q11a	-0.400117315	0.361477211	-1.106894993
trait_Freezings:Block_Tank_GRIM_1_Q12b	-0.724305746	0.373509401	-1.939190136
trait_Freezings:Block_Tank_GRIM_1_Q9a	0.17083584	0.373112896	0.457866351
trait_Freezings:Block_Tank_GRIM_2_R10a	0	NA	NA
trait_Freezings:Block_Tank_GRIM_2_R11a	0.016963716	0.390712428	0.043417396
trait_Freezings:Block_Tank_GRIM_2_R12a	0.07738651	0.410167316	0.188670592
trait_Freezings:Block_Tank_GRIM_2_R9a	0.254843387	0.413290507	0.616620471
trait_Freezings:Block_Tank_SMV_1	0	NA	NA
trait_Freezings:Block_Tank_SMV_2	-0.155604726	0.426413739	-0.364914897
trait_Freezings:Block_Tank_SMV_3	-0.06584416	0.472167887	-0.139450738
trait_Freezings:Block_Tank_SMV_4	-0.126282287	0.476048781	-0.265271737
trait_Freezings:Block_Tank_SMV_5	0.143275872	0.433812306	0.33027157
trait_Freezings:Block_Tank_SMV_6	0.511534679	0.471137344	1.085744284
trait_Freezings:Block_Tank_TMH_P10a	0	NA	NA
trait_Freezings:Block_Tank_TMH_P10b	0.165225025	0.415397993	0.39775114
trait_Freezings:Block_Tank_TMH_P9a	-0.135610898	0.434055949	-0.31242723
trait_Freezings:Block_Tank_TMH_P9b	0.298109006	0.402347534	0.740924152
trait_Shelter:Block_Tank_GRIM_1_Q10b	0	NA	NA
trait_Shelter:Block_Tank_GRIM_1_Q11a	0.489350937	0.311162439	1.572654266
trait_Shelter:Block_Tank_GRIM_1_Q12b	0.747597511	0.321661116	2.324177444
trait_Shelter:Block_Tank_GRIM_1_Q9a	-0.078062071	0.321151933	-0.243068975
trait_Shelter:Block_Tank_GRIM_2_R10a	0	NA	NA
trait_Shelter:Block_Tank_GRIM_2_R11a	-0.484832995	0.336767927	-1.439664993
trait_Shelter:Block_Tank_GRIM_2_R12a	-0.126659394	0.35439551	-0.357395593
trait_Shelter:Block_Tank_GRIM_2_R9a	-0.549100434	0.357856937	-1.534413274
trait_Shelter:Block_Tank_SMV_1	0	NA	NA
trait_Shelter:Block_Tank_SMV_2	0.600369528	0.36839566	1.629686755
trait_Shelter:Block_Tank_SMV_3	-0.265103523	0.407259079	-0.650945643
trait_Shelter:Block_Tank_SMV_4	0.683963965	0.411788268	1.6609603
trait_Shelter:Block_Tank_SMV_5	0.444880941	0.371918427	1.196178811
trait_Shelter:Block_Tank_SMV_6	0.071405707	0.406423315	0.175692939
trait_Shelter:Block_Tank_TMH_P10a	0	NA	NA
trait_Shelter:Block_Tank_TMH_P10b	0.063200673	0.356143104	0.177458646
trait_Shelter:Block_Tank_TMH_P9a	-0.043243158	0.372189578	-0.116185839
trait_Shelter:Block_Tank_TMH_P9b	-0.053659817	0.345097295	-0.155491849

trait_TrackLen:Block_Tank_GRIM_1_Q10b	0	NA	NA
trait_TrackLen:Block_Tank_GRIM_1_Q11a	-0.304819365	0.297173211	-1.02572962
trait_TrackLen:Block_Tank_GRIM_1_Q12b	-0.391683183	0.307108896	-1.275388592
trait_TrackLen:Block_Tank_GRIM_1_Q9a	-0.049246794	0.306727997	-0.160555262
trait_TrackLen:Block_Tank_GRIM_2_R10a	0	NA	NA
trait_TrackLen:Block_Tank_GRIM_2_R11a	-0.227943077	0.321366652	-0.709292875
trait_TrackLen:Block_Tank_GRIM_2_R12a	-0.088515356	0.33768569	-0.262123504
trait_TrackLen:Block_Tank_GRIM_2_R9a	0.413771933	0.340525926	1.215096712
trait_TrackLen:Block_Tank_SMV_1	0	NA	NA
trait_TrackLen:Block_Tank_SMV_2	0.07172328	0.35100213	0.20433859
trait_TrackLen:Block_Tank_SMV_3	0.830134491	0.38846679	2.13695099
trait_TrackLen:Block_Tank_SMV_4	-0.391234986	0.392060998	-0.997893153
trait_TrackLen:Block_Tank_SMV_5	0.003481612	0.356085665	0.009777456
trait_TrackLen:Block_Tank_SMV_6	-0.317219727	0.387621625	-0.818374691
trait_TrackLen:Block_Tank_TMH_P10a	0	NA	NA
trait_TrackLen:Block_Tank_TMH_P10b	-0.048866502	0.340974914	-0.143314069
trait_TrackLen:Block_Tank_TMH_P9a	0.390314208	0.356307506	1.095442003
trait_TrackLen:Block_Tank_TMH_P9b	-0.182850463	0.330314318	-0.553565052
trait_Area:Block_GRIM_1	0	NA	NA
trait_Area:Block_GRIM_2	-0.393422092	0.356403577	-1.103866844
trait_Area:Block_SMV	0	NA	NA
trait_Area:Block_TMH	0.825952278	0.415794525	1.986443373
trait_Exposed:Block_GRIM_1	0	NA	NA
trait_Exposed:Block_GRIM_2	-0.35662529	0.235613498	-1.513602972
trait_Exposed:Block_SMV	0	NA	NA
trait_Exposed:Block_TMH	0.189149312	0.280866974	0.673448036
trait_Freezings:Block_GRIM_1	0	NA	NA
trait_Freezings:Block_GRIM_2	-0.086546509	0.395259161	-0.218961424
trait_Freezings:Block_SMV	0	NA	NA
trait_Freezings:Block_TMH	-0.348715359	0.462719199	-0.75362198
trait_Shelter:Block_GRIM_1	0	NA	NA
trait_Shelter:Block_GRIM_2	0.370258205	0.341520843	1.084145265
trait_Shelter:Block_SMV	0	NA	NA
trait_Shelter:Block_TMH	-0.38955457	0.404137475	-0.96391598
trait_TrackLen:Block_GRIM_1	0	NA	NA
trait_TrackLen:Block_GRIM_2	-0.145026413	0.325413551	-0.445668021
trait_TrackLen:Block_SMV	0	NA	NA
trait_TrackLen:Block_TMH	1.482151104	0.382417175	3.875744081
trait_Area:scale(preTimeNum)	0.009396772	0.059277476	0.158521805
trait_Exposed:scale(preTimeNum)	0.048782362	0.050345292	0.968955785
trait_Freezings:scale(preTimeNum)	-0.010781717	0.069061195	-0.156118316
trait_Shelter:scale(preTimeNum)	0.016690542	0.067831164	0.24606008
trait_TrackLen:scale(preTimeNum)	-0.109382543	0.059821361	-1.828486374
trait_Area:scale(Order, scale = FALSE)	0.007067797	0.021394606	0.330354162
trait_Exposed:scale(Order, scale = FALSE)	-0.006535431	0.017311058	-0.377529268
trait_Freezings:scale(Order, scale = FALSE)	-0.038900161	0.024665519	-1.577106954

trait_Shelter:scale(Order, scale = FALSE)	0.036241736	0.023609921	1.535021507
trait_TrackLen:scale(Order, scale = FALSE)	0.029923146	0.021172128	1.413327296
trait_Area:scale(Replicate, scale = FALSE)	-0.005105541	0.033940513	-0.150426148
trait_Exposed:scale(Replicate, scale = FALSE)	0.0703098	0.028798206	2.441464616
trait_Freezings:scale(Replicate, scale = FALSE)	0.213229851	0.039533576	5.393639275
trait_Shelter:scale(Replicate, scale = FALSE)	-0.075530574	0.038809628	-1.94618134
trait_TrackLen:scale(Replicate, scale = FALSE)	-0.093428349	0.034237535	-2.728828099
trait_Area:SexM	-0.58708839	0.3804024	-1.543335136
trait_Exposed:SexM	-0.315357566	0.254412273	-1.239553276
trait_Freezings:SexM	-0.203943488	0.422652627	-0.482532167
trait_Shelter:SexM	-0.034750514	0.367306643	-0.094608998
trait_TrackLen:SexM	-0.27830016	0.34868365	-0.798145138
trait_Area	1.476743637	0.241152885	6.123682223
trait_Exposed	0.861256719	0.159065896	5.414464954
trait_Freezings	1.28596682	0.267347565	4.810093627
trait_Shelter	0.534378093	0.230743958	2.315892024
trait_TrackLen	1.168066865	0.220013736	5.309063356

(d)

	solution	std error	z ratio
trait_Area:Assay_Bird:StageBin	0	NA	NA
trait_Area:Assay_Fish:StageBin	-0.046416417	0.08146533	-0.569768968
trait_Exposed:Assay_Bird:StageBin	0	NA	NA
trait_Exposed:Assay_Fish:StageBin	0.172559573	0.084847578	2.033759556
trait_Freezings:Assay_Bird:StageBin	0	NA	NA
trait_Freezings:Assay_Fish:StageBin	0.229381489	0.082465069	2.78155942
trait_Shelter:Assay_Bird:StageBin	0	NA	NA
trait_Shelter:Assay_Fish:StageBin	-0.330943241	0.075931466	-4.358446623
trait_TrackLen:Assay_Bird:StageBin	0	NA	NA
trait_TrackLen:Assay_Fish:StageBin	-0.011829921	0.072523743	-0.163117909
trait_Area:Block_Tank_GRIM_1_Q10b	0	NA	NA
trait_Area:Block_Tank_GRIM_1_Q11a	-0.370171501	0.230307218	-1.607294396
trait_Area:Block_Tank_GRIM_1_Q12b	-0.535262109	0.237081834	-2.25771034
trait_Area:Block_Tank_GRIM_1_Q9a	-0.004893664	0.237739337	-0.020584158
trait_Area:Block_Tank_GRIM_2_R10a	0	NA	NA
trait_Area:Block_Tank_GRIM_2_R11a	-0.080991898	0.247755787	-0.326902145
trait_Area:Block_Tank_GRIM_2_R12a	-0.200440205	0.257908728	-0.777174958
trait_Area:Block_Tank_GRIM_2_R9a	0.52318694	0.25919645	2.018495775
trait_Area:Block_Tank_SMV_1	0	NA	NA
trait_Area:Block_Tank_SMV_2	0.140058569	0.267800672	0.522995585
trait_Area:Block_Tank_SMV_3	0.30996092	0.300104334	1.032843863
trait_Area:Block_Tank_SMV_4	-0.082605208	0.297631739	-0.277541662
trait_Area:Block_Tank_SMV_5	0.137421263	0.278622745	0.493216242
trait_Area:Block_Tank_SMV_6	0.027587722	0.300019807	0.091953002
trait_Area:Block_Tank_TMH_P10a	0	NA	NA

trait_Area:Block_Tank_TMH_P10b	-0.156867024	0.266688282	-0.588203662
trait_Area:Block_Tank_TMH_P9a	-0.030561796	0.278861816	-0.109594766
trait_Area:Block_Tank_TMH_P9b	0.022932225	0.25812358	0.088842038
trait_Exposed:Block_Tank_GRIM_1_Q10b	0	NA	NA
trait_Exposed:Block_Tank_GRIM_1_Q11a	-0.30840162	0.22075588	-1.397025621
trait_Exposed:Block_Tank_GRIM_1_Q12b	-0.678986828	0.227146161	-2.989206711
trait_Exposed:Block_Tank_GRIM_1_Q9a	-0.281090199	0.227872081	-1.233543828
trait_Exposed:Block_Tank_GRIM_2_R10a	0	NA	NA
trait_Exposed:Block_Tank_GRIM_2_R11a	0.477307696	0.237499025	2.009724866
trait_Exposed:Block_Tank_GRIM_2_R12a	0.097132176	0.247276583	0.392807821
trait_Exposed:Block_Tank_GRIM_2_R9a	0.4191034	0.248711042	1.685101704
trait_Exposed:Block_Tank_SMV_1	0	NA	NA
trait_Exposed:Block_Tank_SMV_2	-0.164601983	0.256550012	-0.641598033
trait_Exposed:Block_Tank_SMV_3	-0.300555191	0.287897647	-1.04396543
trait_Exposed:Block_Tank_SMV_4	-0.043235368	0.2852102	-0.151591241
trait_Exposed:Block_Tank_SMV_5	-0.089032363	0.266756828	-0.333758515
trait_Exposed:Block_Tank_SMV_6	0.139062762	0.287826979	0.483147069
trait_Exposed:Block_Tank_TMH_P10a	0	NA	NA
trait_Exposed:Block_Tank_TMH_P10b	-0.107722937	0.255323957	-0.421906892
trait_Exposed:Block_Tank_TMH_P9a	-0.041568459	0.267032161	-0.15566836
trait_Exposed:Block_Tank_TMH_P9b	0.167423437	0.247160127	0.677388536
trait_Freezings:Block_Tank_GRIM_1_Q10b	0	NA	NA
trait_Freezings:Block_Tank_GRIM_1_Q11a	-0.181030612	0.276997104	-0.653546947
trait_Freezings:Block_Tank_GRIM_1_Q12b	-0.595383514	0.285418444	-2.086002246
trait_Freezings:Block_Tank_GRIM_1_Q9a	-0.111608398	0.285978397	-0.390268632
trait_Freezings:Block_Tank_GRIM_2_R10a	0	NA	NA
trait_Freezings:Block_Tank_GRIM_2_R11a	0.255085267	0.297918697	0.856224433
trait_Freezings:Block_Tank_GRIM_2_R12a	-0.003113741	0.309849001	-0.010049221
trait_Freezings:Block_Tank_GRIM_2_R9a	0.008350157	0.31094709	0.026853948
trait_Freezings:Block_Tank_SMV_1	0	NA	NA
trait_Freezings:Block_Tank_SMV_2	-0.326259587	0.322672624	-1.011116416
trait_Freezings:Block_Tank_SMV_3	-0.365241819	0.360384546	-1.013478027
trait_Freezings:Block_Tank_SMV_4	-0.129173777	0.358277806	-0.360540828
trait_Freezings:Block_Tank_SMV_5	0.025822341	0.33609775	0.076829852
trait_Freezings:Block_Tank_SMV_6	0.341674395	0.360311207	0.94827579
trait_Freezings:Block_Tank_TMH_P10a	0	NA	NA
trait_Freezings:Block_Tank_TMH_P10b	-0.016078545	0.321727353	-0.049975685
trait_Freezings:Block_Tank_TMH_P9a	-0.133337706	0.336300842	-0.396483413
trait_Freezings:Block_Tank_TMH_P9b	0.267518957	0.31130967	0.859333913
trait_Shelter:Block_Tank_GRIM_1_Q10b	0	NA	NA
trait_Shelter:Block_Tank_GRIM_1_Q11a	0.480091895	0.236535375	2.029683276
trait_Shelter:Block_Tank_GRIM_1_Q12b	0.914428538	0.243632578	3.753309786
trait_Shelter:Block_Tank_GRIM_1_Q9a	0.13402476	0.244189666	0.548855167
trait_Shelter:Block_Tank_GRIM_2_R10a	0	NA	NA
trait_Shelter:Block_Tank_GRIM_2_R11a	-0.351065594	0.254423345	-1.379848199
trait_Shelter:Block_Tank_GRIM_2_R12a	-0.124987627	0.264711364	-0.4721657

trait_Shelter:Block_Tank_GRIM_2_R9a	-0.386999693	0.265802387	-1.45596771
trait_Shelter:Block_Tank_SMV_1	0	NA	NA
trait_Shelter:Block_Tank_SMV_2	0.234624596	0.275334587	0.852143565
trait_Shelter:Block_Tank_SMV_3	-0.029568839	0.307932827	-0.096023665
trait_Shelter:Block_Tank_SMV_4	0.386620912	0.305834967	1.264148819
trait_Shelter:Block_Tank_SMV_5	0.085015136	0.286658779	0.296572588
trait_Shelter:Block_Tank_SMV_6	0.232741534	0.307859045	0.756000311
trait_Shelter:Block_Tank_TMH_P10a	0	NA	NA
trait_Shelter:Block_Tank_TMH_P10b	0.242572872	0.274393163	0.884033953
trait_Shelter:Block_Tank_TMH_P9a	0.13115582	0.286860401	0.457211312
trait_Shelter:Block_Tank_TMH_P9b	-0.052430041	0.265537225	-0.197448928
trait_TrackLen:Block_Tank_GRIM_1_Q10b	0	NA	NA
trait_TrackLen:Block_Tank_GRIM_1_Q11a	-0.429858596	0.2253746	-1.907307195
trait_TrackLen:Block_Tank_GRIM_1_Q12b	-0.458035469	0.232131569	-1.973171816
trait_TrackLen:Block_Tank_GRIM_1_Q9a	-0.058702933	0.232666292	-0.252305277
trait_TrackLen:Block_Tank_GRIM_2_R10a	0	NA	NA
trait_TrackLen:Block_Tank_GRIM_2_R11a	-0.151020979	0.242420226	-0.62297186
trait_TrackLen:Block_Tank_GRIM_2_R12a	-0.144306136	0.252232949	-0.572114531
trait_TrackLen:Block_Tank_GRIM_2_R9a	0.263394201	0.25327898	1.03993707
trait_TrackLen:Block_Tank_SMV_1	0	NA	NA
trait_TrackLen:Block_Tank_SMV_2	0.264502005	0.262325999	1.008295046
trait_TrackLen:Block_Tank_SMV_3	0.61411161	0.29341183	2.093002214
trait_TrackLen:Block_Tank_SMV_4	-0.173259197	0.291395319	-0.594584696
trait_TrackLen:Block_Tank_SMV_5	0.108504481	0.273108702	0.397294117
trait_TrackLen:Block_Tank_SMV_6	-0.308224113	0.29333927	-1.050742757
trait_TrackLen:Block_Tank_TMH_P10a	0	NA	NA
trait_TrackLen:Block_Tank_TMH_P10b	-0.196051834	0.261422132	-0.749943521
trait_TrackLen:Block_Tank_TMH_P9a	0.04160908	0.273301431	0.15224611
trait_TrackLen:Block_Tank_TMH_P9b	-0.237149299	0.252986243	-0.937399978
trait_Area:Block_GRIM_1	0	NA	NA
trait_Area:Block_GRIM_2	-0.292587225	0.24875001	-1.176230002
trait_Area:Block_SMV	0	NA	NA
trait_Area:Block_TMH	0.650991021	0.288235428	2.258539228
trait_Exposed:Block_GRIM_1	0	NA	NA
trait_Exposed:Block_GRIM_2	-0.34091809	0.238514966	-1.429336262
trait_Exposed:Block_SMV	0	NA	NA
trait_Exposed:Block_TMH	-0.168360424	0.277609526	-0.606464866
trait_Freezings:Block_GRIM_1	0	NA	NA
trait_Freezings:Block_GRIM_2	-0.010152278	0.298767611	-0.033980518
trait_Freezings:Block_SMV	0	NA	NA
trait_Freezings:Block_TMH	-0.590584396	0.344303989	-1.715299314
trait_Shelter:Block_GRIM_1	0	NA	NA
trait_Shelter:Block_GRIM_2	0.252327233	0.255271717	0.988465296
trait_Shelter:Block_SMV	0	NA	NA
trait_Shelter:Block_TMH	-0.56677235	0.294800423	-1.922562879
trait_TrackLen:Block_GRIM_1	0	NA	NA

trait_TrackLen:Block_GRIM_2	-0.117916181	0.243240487	-0.484772015
trait_TrackLen:Block_SMV	0	NA	NA
trait_TrackLen:Block_TMH	1.386988094	0.280904319	4.937581951
trait_Area:scale(preTimeNum)	-0.041753733	0.034979416	-1.193665811
trait_Exposed:scale(preTimeNum)	-0.002208688	0.036409713	-0.060662052
trait_Freezings:scale(preTimeNum)	-0.01176336	0.035408424	-0.332219248
trait_Shelter:scale(preTimeNum)	0.060897541	0.032604636	1.867757101
trait_TrackLen:scale(preTimeNum)	-0.085670861	0.031143259	-2.75086367
trait_Area:scale(Order, scale = FALSE)	-0.009459663	0.011297924	-0.837292107
trait_Exposed:scale(Order, scale = FALSE)	-0.036987964	0.011469254	-3.224966752
trait_Freezings:scale(Order, scale = FALSE)	-0.027452271	0.011436254	-2.400459973
trait_Shelter:scale(Order, scale = FALSE)	0.016517544	0.01055033	1.565595062
trait_TrackLen:scale(Order, scale = FALSE)	0.018053867	0.010102288	1.787106761
trait_Area:scale(Replicate, scale = FALSE)	-0.002999472	0.018598139	-0.161278052
trait_Exposed:scale(Replicate, scale = FALSE)	0.090050019	0.019328975	4.658809868
trait_Freezings:scale(Replicate, scale = FALSE)	0.137218633	0.018826736	7.288498217
trait_Shelter:scale(Replicate, scale = FALSE)	-0.056668022	0.017337819	-3.268463072
trait_TrackLen:scale(Replicate, scale = FALSE)	-0.072424478	0.016563329	-4.372579834
trait_Area:SexM	-0.496415978	0.266866898	-1.86016318
trait_Exposed:SexM	0.210461181	0.256874036	0.819316675
trait_Freezings:SexM	0.23963484	0.318776117	0.751733982
trait_Shelter:SexM	-0.000204822	0.272953053	-0.000750392
trait_TrackLen:SexM	-0.461565141	0.260098505	-1.774578219
trait_Area:StageBin	-0.719645712	0.057498568	-12.51588924
trait_Exposed:StageBin	-0.488632902	0.059885774	-8.159415377
trait_Freezings:StageBin	-0.04518896	0.058204188	-0.776386735
trait_Shelter:StageBin	0.575984723	0.053592744	10.74743849
trait_TrackLen:StageBin	-0.623339068	0.051187559	-12.17755014
trait_Area:Assay_Bird	0	NA	NA
trait_Area:Assay_Fish	0.105279933	0.04079309	2.580827603
trait_Exposed:Assay_Bird	0	NA	NA
trait_Exposed:Assay_Fish	-0.42175376	0.042478817	-9.928566532
trait_Freezings:Assay_Bird	0	NA	NA
trait_Freezings:Assay_Fish	0.260098332	0.041293726	6.298737344
trait_Shelter:Assay_Bird	0	NA	NA
trait_Shelter:Assay_Fish	-0.571147784	0.038022606	-15.02126862
trait_TrackLen:Assay_Bird	0	NA	NA
trait_TrackLen:Assay_Fish	0.344231401	0.03631688	9.478551149
trait_Area	1.672313366	0.170307953	9.819349789
trait_Exposed	1.247229705	0.163531987	7.626824114
trait_Freezings	0.788474294	0.204096363	3.863245194
trait_Shelter	1.005075444	0.174537197	5.758517161
trait_TrackLen	1.282569611	0.166315671	7.711658222

Table S3: Effects of 'stage' (pre- to post-) in the control group, where no predator stimulus was applied.

Tank configuration	Behaviour	Effect size	SE	Chisq	P
Bird strike	Area	-1.15	2.2	0.28	0.6
Bird strike	Exposed	-0.38	3.55	0.01	0.91
Bird strike	Freezings	0.19	0.53	0.13	0.72
Bird strike	Shelter	-0.45	0.33	2.6	0.11
Bird strike	Tracklength	3.23	2.19	0.5	0.48
Cichlid reveal	Area	1.32	3.02	0.2	0.66
Cichlid reveal	Exposed	-0.26	0.55	0.24	0.63
Cichlid reveal	Freezings	0.07	0.21	0.13	0.72
Cichlid reveal	Shelter	0.38	0.29	1.77	0.18
Cichlid reveal	Tracklength	-71.25	22.37	8.81	0.003

Appendix S1: ASReml-R code

T.M. Houslay, M. Vierbuchen, A.J. Grimmer, A.J. Young, A.J. Wilson

July 2017

Overview

Below, we provide code to accompany our 2017 Functional Ecology paper, "Testing the stability of behavioural coping style across stress contexts in the Trinidadian guppy". Here we focus on using multivariate mixed models to partition among-individual (co)variation in 5 behavioural traits (measured simultaneously in an open field trial, or OFT). We will demonstrate how to:

- Specify a multivariate mixed model
- Extract the among-individual covariance matrix, known as **I**
- Subject **I** to eigenvector decomposition
- Use bootstrapping methods to estimate 95% confidence intervals around various parameters of interest for **I**
- Compare **I** matrices

Note that we use the `R` interface for `ASReml`, which is commercial software available from VSNi. Similar results can be achieved using the free `R` package `MCMCglmm`, although this requires knowledge of working in a Bayesian framework. We have provided tutorials for multivariate mixed models in both `ASReml-R` and `MCMCglmm` at https://tomhouslay.com/tutorials/, which are associated with an earlier paper *(Houslay & Wilson 2017 Behavioural Ecology)*.

Initialising

Load libraries

Note that you must have the following libraries installed and loaded before running this code.

```
library(asreml)
library(nadiv)
library(mvtnorm)
library(coda)

library(knitr)
library(tidyverse)
```

Data loading / wrangling

The data associated with this paper is available via Dryad.

```
df_oft <- read_csv("Houslayetal_FuncEcol_2017.csv")
```

The data frame comprises the following variables:

- **ID** for each individual
- **Block**
- **Block__Tank**, denoting distinct tanks used over the course of the experiment
- **Assay** indicates tank setup / predator stimulus type
- **Stage**, pre- or post-stimulus
- **StageBin**, as Stage but on a numeric scale

- **preTimeNum**, giving the time (in seconds, from 9am) that the trial began
- **Replicate**, ranging from 1-4
- **Order**, the order in which individuals were assayed within a tank
- **SexM**, numeric variable where 0==Female and 1==Male
- **Mass**, in grams, measured at the end of each trial
- **Area**, calculated as the percent of 1cm x 1cm grid squares entered by the individual during the trial
- **Exposed**, the time (in seconds) the individual spent in the central exposed zone during the trial
- **Freezings**, the number of times the individual 'froze' during the trial
- **Shelter**, the time (in seconds) the individual spent in the shelter during the trial
- **TrackLen**, the total distance travelled (in cm) by the individual during the trial.

We provided the data in raw measurements, but for ease of fitting (and interpreting) multivariate mixed models, we will standardise each behavioural trait by its overall standard deviation (across all setups and contexts). By doing so, we put traits onto similar scales (where 1 unit == 1 standard deviation), but retain any differences in both variation and mean values across contexts.

First, we calculate the global standard deviation for each behaviour:

```
df_stdev <- df_oft %>%
  select(Area, Exposed, Freezings, Shelter, TrackLen) %>%
  gather(Behaviour, Value,
         Area:TrackLen) %>%
  group_by(Behaviour) %>%
  summarise(sdu = sd(Value))

df_stdev
```

```
## # A tibble: 5 x 2
##   Behaviour        sdu
##       <chr>      <dbl>
## 1      Area  13.166626
## 2   Exposed  30.425689
## 3 Freezings   3.516881
## 4   Shelter  42.768470
## 5  TrackLen 157.317042
```

We then divide each observation by the relevant standard deviation:

```
df_oft_sdu <- df_oft %>%  # new data frame will be original after we...
  gather(Behaviour, Value,
         Area:TrackLen) %>%  # convert to 'long' format
  left_join(., df_stdev,
            by = "Behaviour") %>% # join the SD data frame to this by the 'Behaviour' variable
  mutate(Value = Value/sdu) %>% # divide value by its standard deviation
  select(-sdu) %>% # remove the standard deviation variable (no longer needed)
  spread(Behaviour, Value) # return data to the 'wide' format required for multivariate models
```

We also need to create subsets of the data, corresponding to:

- pre-stimulus (both bird strike and cichlid reveal setups)
- post-bird strike
- post-cichlid reveal

```
df_pre <- df_oft_sdu %>%
  filter(Stage == "pre")

df_postbird <- df_oft_sdu %>%
  filter(Stage == "post", Assay == "Bird")
```

```
df_postfish <- df_oft_sdu %>%
  filter(Stage == "post", Assay == "Fish")
```

Model the data

Below, we show the code for the final models in each sequence of context-specific models described in the main text (i.e., 1D, 2D, 3D). These models estimate fully unstructured covariance matrices at both the among-individual and residual levels.

```
# Model 1D
asr_1D <- asreml(cbind(Area,
                       Exposed,
                       Freezings,
                       Shelter,
                       TrackLen) ~
                   trait +
                   trait:(Assay +
                            SexM +
                            scale(Replicate, scale=FALSE) +
                            scale(Order, scale=FALSE) +
                            scale(preTimeNum) +
                            Block +
                            Block_Tank),
                 random =~ ID:us(trait,
                                 init = c(1,
                                          0.1,1,
                                          0.1,0.1,1,
                                          0.1,0.1,0.1,1,
                                          0.1,0.1,0.1,0.1,1)),
                 rcov =~ units:us(trait,
                                  init = rep(0.1,15)),
                 data = df_pre,
                 maxiter = 500)

# Diagnostic plots
hist(residuals(asr_1D))
plot(residuals(asr_1D))
plot(residuals(asr_1D) ~ asr_1D$fitted.values)
qqnorm(resid(asr_1D), main="Q-Q plot for residuals")
```

The variance component summary provides variances and covariances at both the among-individual ('ID:trait!') and residual ('R!') levels, which you can see using the following command (hidden here as it takes up a lot of space!):

```
summary(asr_1D)$varcomp
```

We can repeat these models to partition the among-individual (co)variance in both post-bird strike (2D) and post-cichlid reveal (3D) contexts. Note that we do not need the fixed effect of 'Assay' in these models, as we used that in the pooled pre-stimulus model to allow observations from different setups to have separate means.

```
# Model 2D
asr_2D <- asreml(cbind(Area,
                       Exposed,
```

```
                        Freezings,
                        Shelter,
                        TrackLen) ~
                    trait +
                    trait:(SexM +
                             scale(Replicate, scale=FALSE) +
                             scale(Order, scale=FALSE) +
                             scale(preTimeNum) +
                             Block +
                             Block_Tank),
                  random =~ ID:us(trait,
                                  init = c(1,
                                           0.1,1,
                                           0.1,0.1,1,
                                           0.1,0.1,0.1,1,
                                           0.1,0.1,0.1,0.1,1)),
                  rcov =~ units:us(trait,
                                   init = rep(0.1,15)),
                  data = df_postbird,
                  maxiter = 500)

# Model 3D
asr_3D <- asreml(cbind(Area,
                        Exposed,
                        Freezings,
                        Shelter,
                        TrackLen) ~
                    trait +
                    trait:(SexM +
                             scale(Replicate, scale=FALSE) +
                             scale(Order, scale=FALSE) +
                             scale(preTimeNum) +
                             Block +
                             Block_Tank),
                  random =~ ID:us(trait,
                                  init = c(1,
                                           0.1,1,
                                           0.1,0.1,1,
                                           0.1,0.1,0.1,1,
                                           0.1,0.1,0.1,0.1,1)),
                  rcov =~ units:us(trait,
                                   init = rep(0.1,15)),
                  data = df_postfish,
                  maxiter = 500)
```

Extracting the I matrix

We first define a custom function for reshaping a vector into a full covariance matrix:

```
vecToMat <- function(X, n) {
  S <- diag(n)
  S[upper.tri(S, diag=TRUE)] <- X
  S <- S + t(S) - diag(diag(S))
```

```
  return(S)
}
```

. . . and then extract the among-individual (co)variance estimates from the model summary and create our matrix:

```
# Extract variance components from the model
modpre_df <- data_frame(Var = row.names(summary(asr_1D)$varcomp),
                        Num = summary(asr_1D)$varcomp$component)

# Subset for those where the variable name begins 'ID'
modpre_I_df <- modpre_df %>%
  filter(substring(Var, 1, 2) == "ID")

# Get list of trait names from the model
traitNames <- asr_1D$G.param$ID$trait$levels

# Reform values into covariance matrix
modpre_I_mat <- vecToMat(modpre_I_df$Num, length(traitNames))  ## Second value is number of traits

# Set row and column names
colnames(modpre_I_mat) <- traitNames
rownames(modpre_I_mat) <- traitNames

# Show matrix
kable(modpre_I_mat, digits = 3)
```

	Area	Exposed	Freezings	Shelter	TrackLen
Area	0.182	0.049	-0.030	-0.087	0.109
Exposed	0.049	0.151	0.155	-0.122	-0.005
Freezings	-0.030	0.155	0.235	-0.112	-0.080
Shelter	-0.087	-0.122	-0.112	0.153	-0.081
TrackLen	0.109	-0.005	-0.080	-0.081	0.197

```
# Can also quickly show correlation matrix
kable(cov2cor(modpre_I_mat), digits = 3)
```

	Area	Exposed	Freezings	Shelter	TrackLen
Area	1.000	0.297	-0.144	-0.519	0.574
Exposed	0.297	1.000	0.825	-0.802	-0.026
Freezings	-0.144	0.825	1.000	-0.591	-0.373
Shelter	-0.519	-0.802	-0.591	1.000	-0.465
TrackLen	0.574	-0.026	-0.373	-0.465	1.000

Eigen decomposition

Eigen decomposition is similar to applying a principal components analysis, but here we have isolated the among-individual (co)variance matrix first. As noted in Houslay & Wilson (2017), this enables us to investigate the major axis of among-individual variation (whereas studies that use univariate mixed models on PCA scores from multivariate data are asking whether the major axis of observed behavioural (co)variation is repeatable, where that (co)variation includes both among- and within-individual trait variation).

The output of eigen decomposition is a set of eigenvectors, each of which is associated with:

- An eigenvalue, or the amount of variation associated with that vector
- A 'loading' for each trait, where:
- the value shows how heavily the trait loads
- the sign indicates groupings of traits that load in the same direction

```
# Perform eigen decomposition on pre-stimulus I
I_poolpre_eigen <- eigen(modpre_I_mat)

# View results
I_poolpre_eigen
```

```
## eigen() decomposition
## $values
## [1] 0.455916340 0.365275333 0.079500062 0.013376777 0.004059684
##
## $vectors
##             [,1]        [,2]       [,3]        [,4]       [,5]
## [1,] -0.22423490 -0.56032955  0.7415386 -0.23068443 0.18070633
## [2,] -0.55275677  0.06053543  0.1287940  0.82082935 0.02113501
## [3,] -0.60320962  0.42031140 -0.1228185 -0.43102929 0.50853304
## [4,]  0.52589282  0.22835634  0.2407339  0.28070360 0.73112434
## [5,] -0.06126631 -0.67346713 -0.6004127  0.09188642 0.41683316
```

```
I_poolpre_eigenVals <- I_poolpre_eigen$values
I_poolpre_eigenVecs <- I_poolpre_eigen$vectors

# View proportion of total variation explained by EVs 1 and 2
I_poolpre_eigenVals[1]/sum(I_poolpre_eigenVals)
```

```
## [1] 0.4965715
```

```
I_poolpre_eigenVals[2]/sum(I_poolpre_eigenVals)
```

```
## [1] 0.3978479
```

```
# Associate trait names with the eigen vectors
rownames(I_poolpre_eigenVecs) <- traitNames
I_poolpre_eigenVecs
```

```
##                  [,1]        [,2]       [,3]        [,4]       [,5]
## Area      -0.22423490 -0.56032955  0.7415386 -0.23068443 0.18070633
## Exposed   -0.55275677  0.06053543  0.1287940  0.82082935 0.02113501
## Freezings -0.60320962  0.42031140 -0.1228185 -0.43102929 0.50853304
## Shelter    0.52589282  0.22835634  0.2407339  0.28070360 0.73112434
## TrackLen  -0.06126631 -0.67346713 -0.6004127  0.09188642 0.41683316
```

These steps can be repeated for models 2D and 3D to investigate **I** matrices for post-bird strike and post-cichlid reveal.

Bootstrapping procedure

In our paper, we use a bootstrapping algorithm to put 95% confidence intervals on various estimates (including the trait loadings from the eigenvector decomposition). More importantly, it also enables us to put these confidence intervals on the 'difference matrices' we use to compare context-specific **I** matrices. Note that,

while estimates from the models above should match our results in the paper, there are likely to be small differences in the bootstrapped CIs (as these are calculated from random draws from a specified distribution).

We need the estimates of our three covariance matrices, and for each of these we also need the sampling covariances. Together, these will allow us to specify a multivariate normal distribution from which we can take sample random draws.

```
# Pooled pre

# Get average information matrix
modpre_ai <- as.numeric(asr_1D$ai)

# Find the sampling (co)-variances
modpre_VC <- aiFun(asr_1D, modpre_ai)

# Subset for I (the section of the ai matrix concerned with ID - numbers hard-coded here)
modpre_I_VC <- modpre_VC[1:15,1:15]

# Get estimates of covariances
modpre_I_ests <- modpre_I_df$Num

# Eigenvectors 1 and 2
modpre_I_PC1 <- eigen(modpre_I_mat)$vectors[,1]
modpre_I_PC2 <- eigen(modpre_I_mat)$vectors[,2]



# Post-bird strike

# Extract variance components from the model
modbird_df <- data_frame(Var = row.names(summary(asr_2D)$varcomp),
                        Num = summary(asr_2D)$varcomp$component)

# Subset for those where the variable name begins 'ID'
modbird_I_df <- modbird_df %>%
  filter(substring(Var, 1, 2) == "ID")

# Get list of trait names from the model
traitNames <- asr_2D$G.param$ID$trait$levels

# Reform values into covariance matrix
modbird_I_mat <- vecToMat(modbird_I_df$Num, length(traitNames))  ## Second value is number of traits

# Get average information matrix
modbird_ai <- as.numeric(asr_2D$ai)

# Find the sampling (co)-variances
modbird_VC <- aiFun(asr_2D, modbird_ai)

# Subset for I (the section of the ai matrix concerned with ID - numbers hard-coded here)
modbird_I_VC <- modbird_VC[1:15,1:15]

# Get estimates of covariances
modbird_I_ests <- modbird_I_df$Num
```

```
# Eigenvectors 1 and 2
modbird_I_PC1 <- eigen(modbird_I_mat)$vectors[,1]
modbird_I_PC2 <- eigen(modbird_I_mat)$vectors[,2]



# Post-fish reveal

# Extract variance components from the model
modfish_df <- data_frame(Var = row.names(summary(asr_3D)$varcomp),
                        Num = summary(asr_3D)$varcomp$component)

# Subset for those where the variable name begins 'ID'
modfish_I_df <- modfish_df %>%
  filter(substring(Var, 1, 2) == "ID")

# Get list of trait names from the model
traitNames <- asr_3D$G.param$ID$trait$levels

# Reform values into covariance matrix
modfish_I_mat <- vecToMat(modfish_I_df$Num, length(traitNames))  ## Second value is number of traits

# Get average information matrix
modfish_ai <- as.numeric(asr_3D$ai)

# Find the sampling (co)-variances
modfish_VC <- aiFun(asr_3D, modfish_ai)

# Subset for I (the section of the ai matrix concerned with ID - numbers hard-coded here)
modfish_I_VC <- modfish_VC[1:15,1:15]

# Get estimates of covariances
modfish_I_ests <- modfish_I_df$Num

# Eigenvectors 1 and 2
modfish_I_PC1 <- eigen(modfish_I_mat)$vectors[,1]
modfish_I_PC2 <- eigen(modfish_I_mat)$vectors[,2]
```

We also need to set up a number of empty vectors that can be populated within the bootstrapping algorithm:

```
# Set the number of iterations for the bootstrap
N <- 5000

# I matrices
boot_I_pre <- numeric()
boot_I_postbird <- numeric()
boot_I_postfish <- numeric()

# I correlation
boot_I_pre_cor <- numeric()
boot_I_postbird_cor <- numeric()
boot_I_postfish_cor <- numeric()

## Eigen analysis
```

```
boot_loading_pre_1 <- numeric()
boot_loading_postbird_1 <- numeric()
boot_loading_postfish_1 <- numeric()

boot_loading_pre_2 <- numeric()
boot_loading_postbird_2 <- numeric()
boot_loading_postfish_2 <- numeric()
```

Next we perform the bootstrap algorithm. For 5000 replicates, we sample a matrix draw from each of the pre-stimulus, post-bird strike, and post-cichlid reveal multivariate normal distributions. We store these covariance matrix draws, along with correlation matrix versions and trait loadings for the first 2 eigenvectors.

```
for (i in 1:N)
{

  ## Sample from multivariate normal for each I matrix
  draw_I_pre <- rmvnorm(1, modpre_I_ests, modpre_I_VC)
  draw_I_postbird <- rmvnorm(1, modbird_I_ests, modbird_I_VC)
  draw_I_postfish <- rmvnorm(1, modfish_I_ests, modfish_I_VC)

  ## Store I sample
  boot_I_pre <- rbind(boot_I_pre, draw_I_pre)
  boot_I_postbird <- rbind(boot_I_postbird, draw_I_postbird)
  boot_I_postfish <- rbind(boot_I_postfish, draw_I_postfish)

  ## Convert samples to matrix form (to get correlations easily)
  draw_I_pre_mat <- vecToMat(draw_I_pre, 5)
  draw_I_postbird_mat <- vecToMat(draw_I_postbird, 5)
  draw_I_postfish_mat <- vecToMat(draw_I_postfish, 5)

  ## Calculate and store bootstrapped correlations

  # ..calculate
  draw_I_pre_cormat <- cov2cor(draw_I_pre_mat)
  draw_I_pre_cor <- draw_I_pre_cormat[upper.tri(draw_I_pre_cormat, diag=TRUE)]
  draw_I_postbird_cormat <- cov2cor(draw_I_postbird_mat)
  draw_I_postbird_cor <- draw_I_postbird_cormat[upper.tri(draw_I_postbird_cormat, diag=TRUE)]
  draw_I_postfish_cormat <- cov2cor(draw_I_postfish_mat)
  draw_I_postfish_cor <- draw_I_postfish_cormat[upper.tri(draw_I_postfish_cormat, diag=TRUE)]

  # ..store
  boot_I_pre_cor <- rbind(boot_I_pre_cor, draw_I_pre_cor)
  boot_I_postbird_cor <- rbind(boot_I_postbird_cor, draw_I_postbird_cor)
  boot_I_postfish_cor <- rbind(boot_I_postfish_cor, draw_I_postfish_cor)


  ## Eigenvector decomposition
  eigen_pre <- eigen(draw_I_pre_mat)
  eigen_postbird <- eigen(draw_I_postbird_mat)
  eigen_postfish <- eigen(draw_I_postfish_mat)


  ## Get trait loadings for eigens 1 and 2 (PC1-2)
  draw_I_pre_PC1 <- eigen_pre$vectors[,1]
```

```
draw_I_postbird_PC1 <- eigen_postbird$vectors[,1]
draw_I_postfish_PC1 <- eigen_postfish$vectors[,1]

draw_I_pre_PC2 <- eigen_pre$vectors[,2]
draw_I_postbird_PC2 <- eigen_postbird$vectors[,2]
draw_I_postfish_PC2 <- eigen_postfish$vectors[,2]

##
# Draws aren't necessarily done in the same 'space' as original eigen decomp of I matrix
# - ie, the sign is just used to group traits that load in the same direction, but
#         the sign itself is assigned arbitrarily
# - to make sure we are putting everything in the same space,
#      if angle between draw and mean is >90 then flip signs on all loadings
##

## Pre (pooled)

## PC 1
theta_pre_PC1 <- acos(sum(modpre_I_PC1*draw_I_pre_PC1) /
                     (sqrt(sum(modpre_I_PC1 * modpre_I_PC1)) *
                        sqrt(sum(draw_I_pre_PC1 * draw_I_pre_PC1))))
### convert to degrees
theta_pre_deg1 <- (180/pi)*theta_pre_PC1

#if statement flips signs of trait loadings on this draw if angle >90
if (theta_pre_deg1 > 90) {
  draw_I_pre_PC1 <- draw_I_pre_PC1*-1
} else {
  draw_I_pre_PC1 < -draw_I_pre_PC1
}

## PC 2
theta_pre_PC2 <- acos(sum(modpre_I_PC2*draw_I_pre_PC2) /
                     (sqrt(sum(modpre_I_PC2 * modpre_I_PC2)) *
                        sqrt(sum(draw_I_pre_PC2 * draw_I_pre_PC2))))
### convert to degrees
theta_pre_deg2 <- (180/pi)*theta_pre_PC2

#if statement flips signs of trait loadings on this draw if angle >90
if (theta_pre_deg2 > 90) {
  draw_I_pre_PC2 <- draw_I_pre_PC2*-1
} else {
  draw_I_pre_PC2 < -draw_I_pre_PC2
}

## Store trait loadings
boot_loading_pre_1 <- rbind(boot_loading_pre_1, draw_I_pre_PC1)
boot_loading_pre_2 <- rbind(boot_loading_pre_2, draw_I_pre_PC2)




## Postbird
```

```
## PC 1
theta_postbird_PC1 <- acos(sum(modbird_I_PC1*draw_I_postbird_PC1) /
                     (sqrt(sum(modbird_I_PC1 * modbird_I_PC1)) *
                        sqrt(sum(draw_I_postbird_PC1 * draw_I_postbird_PC1))))
### convert to degrees
theta_postbird_deg1 <- (180/pi)*theta_postbird_PC1

#if statement flips signs of trait loadings on this draw if angle >90
if (theta_postbird_deg1 > 90) {
  draw_I_postbird_PC1 <- draw_I_postbird_PC1*-1
} else {
  draw_I_postbird_PC1 <- draw_I_postbird_PC1
}

## PC 2
theta_postbird_PC2 <- acos(sum(modbird_I_PC1*draw_I_postbird_PC2) /
                     (sqrt(sum(modbird_I_PC2 * modbird_I_PC2)) *
                        sqrt(sum(draw_I_postbird_PC2 * draw_I_postbird_PC2))))
### convert to degrees
theta_postbird_deg2 <- (180/pi)*theta_postbird_PC2

#if statement flips signs of trait loadings on this draw if angle >90
if (theta_postbird_deg2 > 90) {
  draw_I_postbird_PC2 <- draw_I_postbird_PC2*-1
} else {
  draw_I_postbird_PC2 <- draw_I_postbird_PC2
}

## Store trait loadings
boot_loading_postbird_1 <- rbind(boot_loading_postbird_1, draw_I_postbird_PC1)
boot_loading_postbird_2 <- rbind(boot_loading_postbird_2, draw_I_postbird_PC2)



## Postfish

## PC 1
theta_postfish_PC1 <- acos(sum(modfish_I_PC1*draw_I_postfish_PC1) /
                     (sqrt(sum(modfish_I_PC1 * modfish_I_PC1)) *
                        sqrt(sum(draw_I_postfish_PC1 * draw_I_postfish_PC1))))
### convert to degrees
theta_postfish_deg1 <- (180/pi)*theta_postfish_PC1

#if statement flips signs of trait loadings on this draw if angle >90
if (theta_postfish_deg1 > 90) {
  draw_I_postfish_PC1 <- draw_I_postfish_PC1*-1
} else {
  draw_I_postfish_PC1 <- draw_I_postfish_PC1
}

## PC 2
theta_postfish_PC2 <- acos(sum(modfish_I_PC1*draw_I_postfish_PC2) /
                     (sqrt(sum(modfish_I_PC2 * modfish_I_PC2)) *
```

```
                          sqrt(sum(draw_I_postfish_PC2 * draw_I_postfish_PC2))))
  ### convert to degrees
  theta_postfish_deg2 <- (180/pi)*theta_postfish_PC2

  #if statement flips signs of trait loadings on this draw if angle >90
  if (theta_postfish_deg2 > 90) {
    draw_I_postfish_PC2 <- draw_I_postfish_PC2*-1
  } else {
    draw_I_postfish_PC2 <- draw_I_postfish_PC2
  }

  ## Store trait loadings
  boot_loading_postfish_1 <- rbind(boot_loading_postfish_1, draw_I_postfish_PC1)
  boot_loading_postfish_2 <- rbind(boot_loading_postfish_2, draw_I_postfish_PC2)


}
```

Confidence intervals on I matrices

Here we demonstrate how to find the 95% confidence intervals on variance, covariance and correlation estimates for the pre-stimulus **I** matrix (and output this in a readable format):

```
## Get upper and lower bounds of I matrix estimates

modpre_I_mat_lower <- vecToMat(as.numeric(HPDinterval(as.mcmc(boot_I_pre), prob=0.95)[,'lower']),5)
modpre_I_mat_upper <- vecToMat(as.numeric(HPDinterval(as.mcmc(boot_I_pre), prob=0.95)[,'upper']),5)


modpre_I_mat_cor_lower <- vecToMat(c(HPDinterval(as.mcmc(boot_I_pre_cor[,1]), prob=0.95)[,'lower'],
           HPDinterval(as.mcmc(boot_I_pre_cor[,2]), prob=0.95)[,'lower'],
           HPDinterval(as.mcmc(boot_I_pre_cor[,3]), prob=0.95)[,'lower'],
           HPDinterval(as.mcmc(boot_I_pre_cor[,4]), prob=0.95)[,'lower'],
           HPDinterval(as.mcmc(boot_I_pre_cor[,5]), prob=0.95)[,'lower'],
           HPDinterval(as.mcmc(boot_I_pre_cor[,6]), prob=0.95)[,'lower'],
           HPDinterval(as.mcmc(boot_I_pre_cor[,7]), prob=0.95)[,'lower'],
           HPDinterval(as.mcmc(boot_I_pre_cor[,8]), prob=0.95)[,'lower'],
           HPDinterval(as.mcmc(boot_I_pre_cor[,9]), prob=0.95)[,'lower'],
           HPDinterval(as.mcmc(boot_I_pre_cor[,10]), prob=0.95)[,'lower'],
           HPDinterval(as.mcmc(boot_I_pre_cor[,11]), prob=0.95)[,'lower'],
           HPDinterval(as.mcmc(boot_I_pre_cor[,12]), prob=0.95)[,'lower'],
           HPDinterval(as.mcmc(boot_I_pre_cor[,13]), prob=0.95)[,'lower'],
           HPDinterval(as.mcmc(boot_I_pre_cor[,14]), prob=0.95)[,'lower'],
           HPDinterval(as.mcmc(boot_I_pre_cor[,15]), prob=0.95)[,'lower']),
         5)

modpre_I_mat_cor_upper <- vecToMat(c(HPDinterval(as.mcmc(boot_I_pre_cor[,1]), prob=0.95)[,'lower'],
           HPDinterval(as.mcmc(boot_I_pre_cor[,2]), prob=0.95)[,'upper'],
           HPDinterval(as.mcmc(boot_I_pre_cor[,3]), prob=0.95)[,'upper'],
           HPDinterval(as.mcmc(boot_I_pre_cor[,4]), prob=0.95)[,'upper'],
           HPDinterval(as.mcmc(boot_I_pre_cor[,5]), prob=0.95)[,'upper'],
           HPDinterval(as.mcmc(boot_I_pre_cor[,6]), prob=0.95)[,'upper'],
           HPDinterval(as.mcmc(boot_I_pre_cor[,7]), prob=0.95)[,'upper'],
```

```
          HPDinterval(as.mcmc(boot_I_pre_cor[,8]), prob=0.95)[,'upper'],
          HPDinterval(as.mcmc(boot_I_pre_cor[,9]), prob=0.95)[,'upper'],
          HPDinterval(as.mcmc(boot_I_pre_cor[,10]), prob=0.95)[,'upper'],
          HPDinterval(as.mcmc(boot_I_pre_cor[,11]), prob=0.95)[,'upper'],
          HPDinterval(as.mcmc(boot_I_pre_cor[,12]), prob=0.95)[,'upper'],
          HPDinterval(as.mcmc(boot_I_pre_cor[,13]), prob=0.95)[,'upper'],
          HPDinterval(as.mcmc(boot_I_pre_cor[,14]), prob=0.95)[,'upper'],
          HPDinterval(as.mcmc(boot_I_pre_cor[,15]), prob=0.95)[,'upper']),
        5)



modpre_I_mat_cor <- cov2cor(modpre_I_mat)

I_error_pre <- matrix(NA, 5, 5)

for(i in 1:5){
  for(j in 1:5){
    if(j > i){
      I_error_pre[i,j] <- paste(round(modpre_I_mat_cor[i,j],digits=2),
                                " (",
                                round(modpre_I_mat_cor_lower[i,j],digits=2),
                                ",",
                                round(modpre_I_mat_cor_upper[i,j],digits=2),
                                ")",
                                sep = "")
    } else {
      I_error_pre[i,j] <- paste(round(modpre_I_mat[i,j],digits=2),
                                " (",
                                round(modpre_I_mat_lower[i,j],digits=2),
                                ",",
                                round(modpre_I_mat_upper[i,j],digits=2),
                                ")",
                                sep = "")
    }
  }
}

colnames(I_error_pre) <- traitNames
rownames(I_error_pre) <- traitNames

kable(I_error_pre)
```

	Area	Exposed	Freezings	Shelter	TrackLen
Area	0.18 (0.1,0.26)	0.3 (-0.06,0.61)	-0.14 (-0.44,0.2)	-0.52 (-0.76,-0.27)	0.57 (0.34,0.78)
Exposed	0.05 (-0.01,0.11)	0.15 (0.08,0.22)	0.82 (0.67,1)	-0.8 (-0.96,-0.65)	-0.03 (-0.35,0.32)
Freezings	-0.03 (-0.09,0.03)	0.16 (0.08,0.22)	0.24 (0.14,0.34)	-0.59 (-0.8,-0.38)	-0.37 (-0.62,-0.1)
Shelter	-0.09 (-0.14,-0.03)	-0.12 (-0.18,-0.07)	-0.11 (-0.17,-0.05)	0.15 (0.09,0.21)	-0.47 (-0.69,-0.23)
TrackLen	0.11 (0.05,0.17)	0 (-0.06,0.05)	-0.08 (-0.15,-0.02)	-0.08 (-0.14,-0.03)	0.2 (0.12,0.28)

Confidence intervals on trait loadings for eigen decomposition

```
df_vis_eigen_poolpre <- data.frame(Eigen = 1:2,
                                   as.data.frame(rbind(I_poolpre_eigenVecs[,1],
                                                       I_poolpre_eigenVecs[,2]))) %>%
  gather(., Trait, Value, Area:TrackLen)

df_vis_eigen_poolpre$lower <- c(HPDinterval(as.mcmc(boot_loading_pre_1[,1]), 0.95)[,"lower"],
                                HPDinterval(as.mcmc(boot_loading_pre_2[,1]), 0.95)[,"lower"],
                                HPDinterval(as.mcmc(boot_loading_pre_1[,2]), 0.95)[,"lower"],
                                HPDinterval(as.mcmc(boot_loading_pre_2[,2]), 0.95)[,"lower"],
                                HPDinterval(as.mcmc(boot_loading_pre_1[,3]), 0.95)[,"lower"],
                                HPDinterval(as.mcmc(boot_loading_pre_2[,3]), 0.95)[,"lower"],
                                HPDinterval(as.mcmc(boot_loading_pre_1[,4]), 0.95)[,"lower"],
                                HPDinterval(as.mcmc(boot_loading_pre_2[,4]), 0.95)[,"lower"],
                                HPDinterval(as.mcmc(boot_loading_pre_1[,5]), 0.95)[,"lower"],
                                HPDinterval(as.mcmc(boot_loading_pre_2[,5]), 0.95)[,"lower"])


df_vis_eigen_poolpre$upper <- c(HPDinterval(as.mcmc(boot_loading_pre_1[,1]), 0.95)[,"upper"],
                                HPDinterval(as.mcmc(boot_loading_pre_2[,1]), 0.95)[,"upper"],
                                HPDinterval(as.mcmc(boot_loading_pre_1[,2]), 0.95)[,"upper"],
                                HPDinterval(as.mcmc(boot_loading_pre_2[,2]), 0.95)[,"upper"],
                                HPDinterval(as.mcmc(boot_loading_pre_1[,3]), 0.95)[,"upper"],
                                HPDinterval(as.mcmc(boot_loading_pre_2[,3]), 0.95)[,"upper"],
                                HPDinterval(as.mcmc(boot_loading_pre_1[,4]), 0.95)[,"upper"],
                                HPDinterval(as.mcmc(boot_loading_pre_2[,4]), 0.95)[,"upper"],
                                HPDinterval(as.mcmc(boot_loading_pre_1[,5]), 0.95)[,"upper"],
                                HPDinterval(as.mcmc(boot_loading_pre_2[,5]), 0.95)[,"upper"])


ggplot(df_vis_eigen_poolpre, aes(x = Trait, y = Value)) +
  geom_hline(yintercept = 0,
             linetype = 2,
             colour = 'grey75') +
  geom_hline(yintercept = -0.5,
             linetype = 3,
             colour = 'grey90') +
  geom_hline(yintercept = 0.5,
             linetype = 3,
             colour = 'grey90') +
    geom_hline(yintercept = -1,
             linetype = 3,
             colour = 'grey90') +
  geom_hline(yintercept = 1,
             linetype = 3,
             colour = 'grey90') +
  geom_pointrange(aes(ymin = lower,
                      ymax = upper),
                  colour = "grey40") +
  labs(x = "Behaviour",
       y = "Trait loading") +
  scale_x_discrete(limits = rev(traitNames)) +
```

```
  ylim(c(-1,1)) +
  coord_flip() +
  facet_grid(. ~ Eigen, labeller = label_both) +
  theme_classic()
```

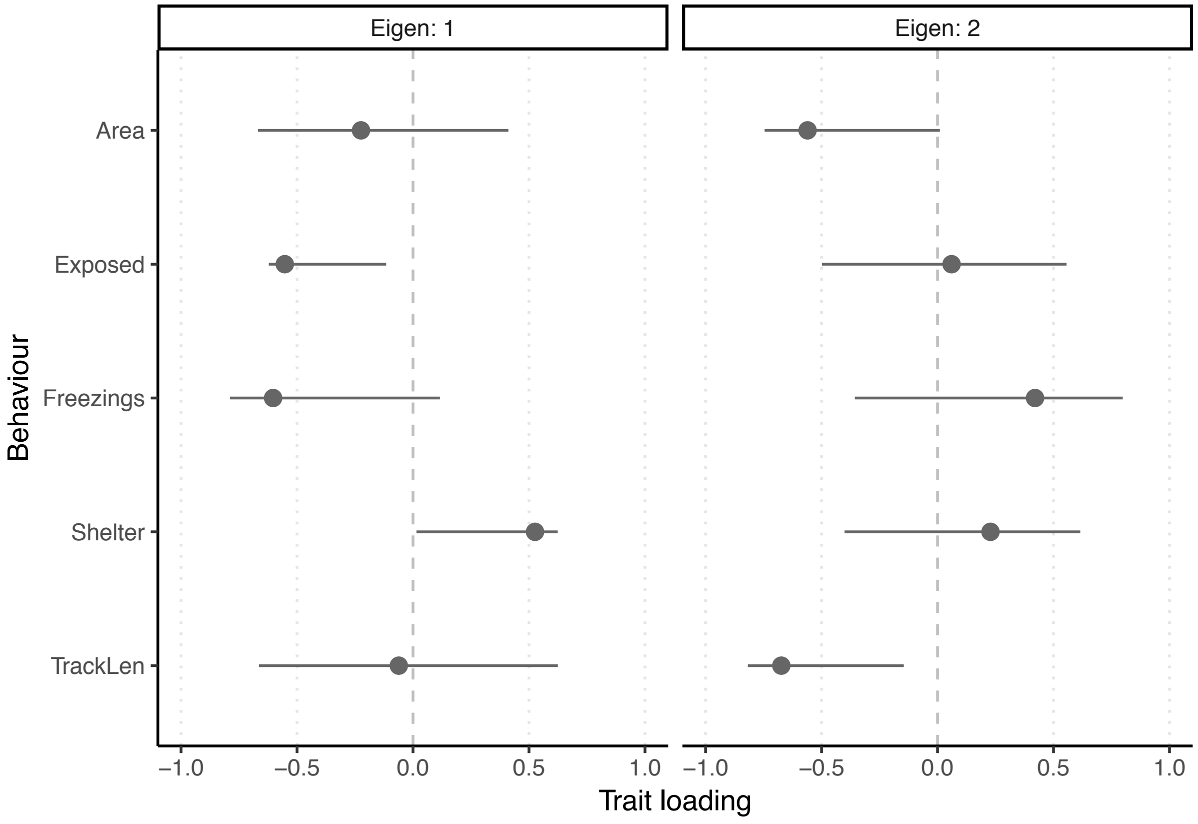

Difference matrix

Here, we show how to demonstrate the difference between the (pooled) pre-stimulus **I** matrix and the post-bird strike **I** matrix. The estimates of the differences are given by simply subtracting I_{pre} from $I_{post\text{-}bird}$; the confidence intervals are estimated by subtracting the set of pre-stimulus bootstrap draws from those of post-bird strike and then finding the 95% confidence limits of the resultant distribution.

```
# Get difference in estimates from respective I matrices
I_diff_mat_pre_postbird <- modbird_I_mat - modpre_I_mat

# Subtract one set of bootstrap draws from the other
I_bootdiff_pre_postbird <- boot_I_postbird - boot_I_pre

# Get upper and lower confidence intervals from this set of difference values
I_diff_mat_pre_postbird_lower <- vecToMat(as.numeric(HPDinterval(as.mcmc(I_bootdiff_pre_postbird),
                                                      prob=0.95)[,'lower']),5)
I_diff_mat_pre_postbird_upper <- vecToMat(as.numeric(HPDinterval(as.mcmc(I_bootdiff_pre_postbird),
                                                      prob=0.95)[,'upper']),5)


## Reform into readable matrix
```

```
I_diff_error_pre_postbird <- matrix(NA, 5, 5)

for(i in 1:5){
  for(j in 1:5){
    I_diff_error_pre_postbird[i,j] <- paste(round(I_diff_mat_pre_postbird[i,j],digits=2),
                                              " (",
                                              round(I_diff_mat_pre_postbird_lower[i,j],digits=2),
                                              ",",
                                              round(I_diff_mat_pre_postbird_upper[i,j],digits=2),
                                            ")",
                                            sep = "")
  }
}

# Associate trait names
colnames(I_diff_error_pre_postbird) <- traitNames
rownames(I_diff_error_pre_postbird) <- traitNames

# Print matrix
kable(I_diff_error_pre_postbird)
```

	Area	Exposed	Freezings	Shelter	TrackLen
Area	-0.1 (-0.2,0.01)	0.01 (-0.09,0.11)	0.06 (-0.03,0.15)	0.02 (-0.07,0.11)	-0.05 (-0.14,0.03)
Exposed	0.01 (-0.09,0.11)	0.13 (-0.03,0.3)	0.05 (-0.08,0.19)	-0.06 (-0.2,0.06)	0.02 (-0.07,0.1)
Freezings	0.06 (-0.03,0.15)	0.05 (-0.08,0.19)	-0.06 (-0.21,0.08)	0.01 (-0.11,0.12)	0.05 (-0.04,0.14)
Shelter	0.02 (-0.07,0.11)	-0.06 (-0.2,0.06)	0.01 (-0.11,0.12)	0 (-0.12,0.13)	0.01 (-0.08,0.1)
TrackLen	-0.05 (-0.14,0.03)	0.02 (-0.07,0.1)	0.05 (-0.04,0.14)	0.01 (-0.08,0.1)	-0.08 (-0.18,0.03)
